# Supplementary material for: Molecular-level architecture of Chlamydomonas reinhardtii’s glycoprotein-rich cell wall
Source: Nat Commun. 2024 Feb 2;15:986. doi: 10.1038/s41467-024-45246-7 (PMC10837150; doi:10.1038/s41467-024-45246-7)
Supplement: Supplementary file 1 — Supplementary Information [file 41467_2024_45246_MOESM1_ESM.pdf]

Supplementary Information

Molecular-Level Architecture of *Chlamydomonas reinhardtii's* Glycoprotein-Rich Cell Wall

Alexandre Poulhazan[1], Alexandre A. Arnold[1], Frederic Mentink-Vigier[2], Artur Muszyński[3], Parastoo Azadi[3], Adnan Halim[4], Sergey Y. Vakhrushev[4], Hiren Jitendra Joshi[4], Tuo Wang[5]*, Dror E. Warschawski[6]* and Isabelle Marcotte[1]*

[1] Department of Chemistry, University of Quebec at Montreal, Montreal, H2X 2J6, Canada

[2] National High Magnetic Field Laboratory, Florida State University, Tallahassee, FL 32310, USA

[3] Complex Carbohydrate Research Center, University of Georgia, Athens, GA 30602, USA

[4] Copenhagen Center for Glycomics, University of Copenhagen, Copenhagen, Denmark

[5] Department of Chemistry, Michigan State University, East Lansing, MI 48824, USA

[6] Laboratoire des Biomolécules, LBM, CNRS UMR 7203, Sorbonne Université, École Normale Supérieure, PSL University, 75005 Paris, France

* Corresponding authors:

marcotte.isabelle@uqam.ca, dror.warschawski@sorbonne-universite.fr, wangtuo1@msu.edu

Table of content

Supplementary Figure 1. MAS-DNP allows high sensitivity enhancement but loss in resolution 4
Supplementary Figure 2. Comparison of bald2 cell wall and stda 6-1 whole cell at 800 MHz 5
Supplementary Figure 3. Comparison of the cell wall spectra of *wt* and *bald2* strains 6
Supplementary Figure 4. Complete DP-INADEQUATE spectra of *C. reinhardtii's* cell wall 7
Supplementary Figure 5. Protein carbonyl assignment of *C. reinhardtii's* cell wall 8
Supplementary Figure 6. Protein Cα assignment of *C. reinhardtii's* cell wall 9
Supplementary Figure 7. Protein carbonyl assignment of *C. reinhardtii's* cell wall 10
Supplementary Figure 8. ssNMR of *C. reinhardtii* cell wall at cryogenic and 283 K 11
Supplementary Figure 9. Effect of deglycosylation on *C. reinhardtii* cell-wall extract 12
Supplementary Figure 10. Map of the resolved glycan-Hyp or -Thr spatial contacts 13
Supplementary Figure 11. DARR/PDSD build-up spectra 14
Supplementary Figure 12. DARR/PDSD 2D spectral editing 15
Supplementary Figure 13. NCaCx MAS-DNP and higher field spectra 16
Supplementary Figure 14. 2D ^{13}C-^{13}C DARR water edition applied to *C. reinhardtii* cell wall 17
Supplementary Figure 15. DNP build-up of *C. reinhardtii's* cell wall extract 18
Supplementary Figure 16. 1D experiments revealing dynamics in *C. reinhardtii* cell wall 19
Supplementary Figure 17. CP build-up in *C. reinhardtii's* constituents 20
Supplementary Figure 18. Relaxation measurement of *C. reinhardtii's* cell wall 21
Supplementary Figure 19. Uncropped SD-PAGE gels 22
Supplementary Figure 20. Representative MS/MS spectra of glycopeptides 23
Supplementary Table 1. *wt* and *bald2* cell-wall glycan and amino acid composition 24
Supplementary Table 2. Glycosyl linkages determined by GC-MS in different samples 25
Supplementary Table 3. Full ^{13}C ssNMR glycan assignment of *C. reinhardtii bald2*'s cell wall 26
Supplementary Table 4. ^{13}C ssNMR glycan assignment and comparison with literature values 27
Supplementary Table 5. Glycan integrals used for ssNMR quantification 31
Supplementary Table 6. Results of the ssNMR-based glycan quantification 36
Supplementary Table 7. ^{13}C glycan composition of different intact *C. reinhardtii* strains 37
Supplementary Table 8. Glycan content determined by GC-MS 38
Supplementary Table 9. ^{13}C ssNMR amino acid assignment of *C. reinhardtii bald2* cell wall 39
Supplementary Table 10. Integrals of the amino acid used for their quantification 41
Supplementary Table 11. Results of the amino acid quantification 47
Supplementary Table 12. Amino acid composition of *wt* and *bald2 C. reinhardtii* strains 49
Supplementary Table 13. Protein-glycan contacts detected by MAS-DNP in the cell wall 50

Supplementary Table 14. Glycoproteomics and identification of hexose modified proteins/peptides **51**
Supplementary Table 15. ^{13}C DARR cross-peak intensities and calculated hydration level **53**
Supplementary Table 16. Resonance-specific ^{13}C intensity DNP build-up **59**
Supplementary Table 17. Assignment of 1D INEPT and CP ^{13}C ssNMR peaks **60**
Supplementary Table 18. ^{13}C resonance CP intensity build-up in *C. reinhardtii*'s cell wall extract **61**
Supplementary Table 19. ^{13}C resonance CP intensity build-up in *C. reinhardtii*'s starch **62**
Supplementary Table 20. List of experiments performed in this work and acquisition time **63**
Supplementary Table 21. List of replicates used for ssNMR in this study **65**
References **66**

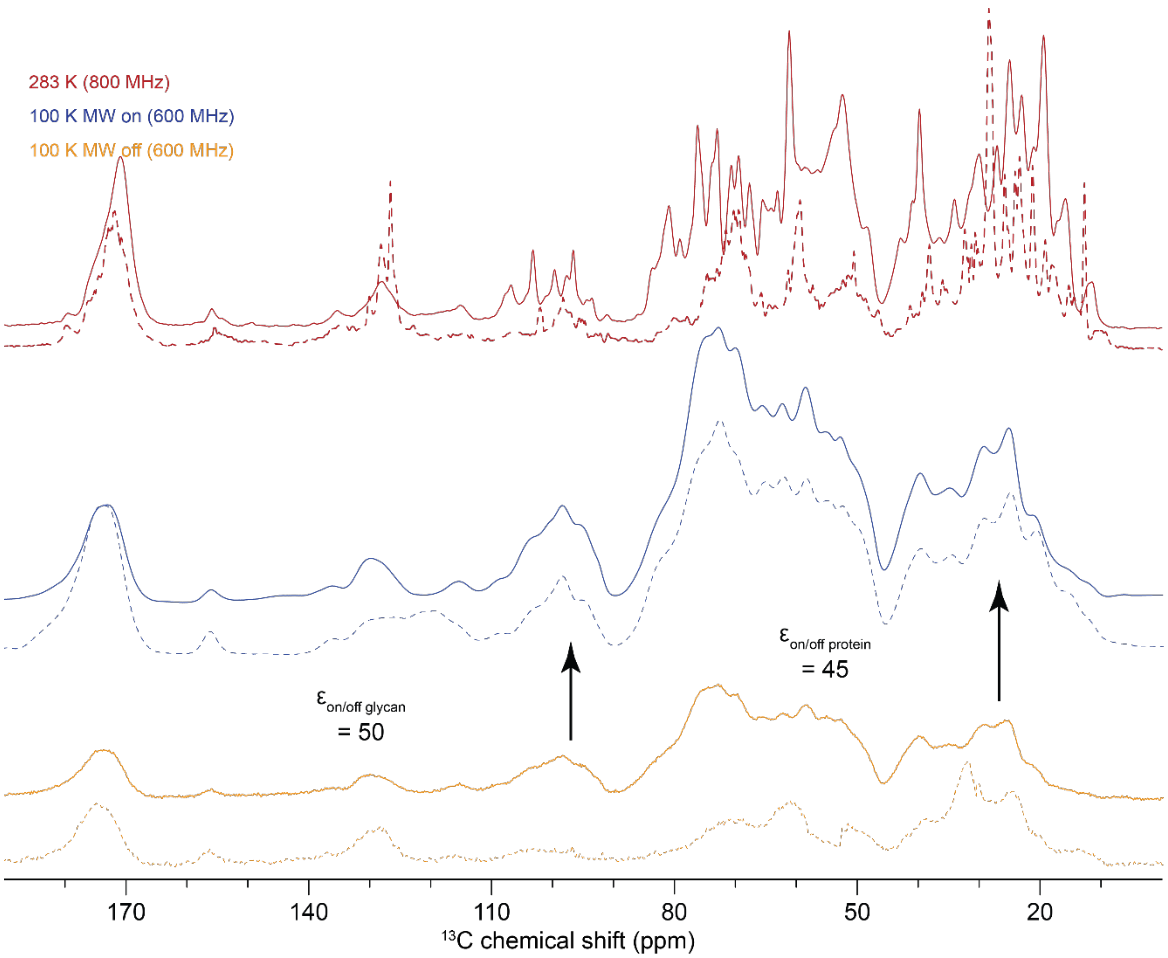

Supplementary Figure 1. MAS-DNP allows high sensitivity enhancement but loss in resolution. 1D ^{13}C CP spectra of *C. reinhardtii* cell-wall extract and whole cell (dashed line) at 600 MHz and 100 K with (microwaves signal enhancement ON, blue spectra) and without (microwave signal enhancement OFF, orange spectra) MAS-DNP enhancement (cryogenic temperature needed for MAS-DNP enhancement), and at 800 MHz and 283 K (red).

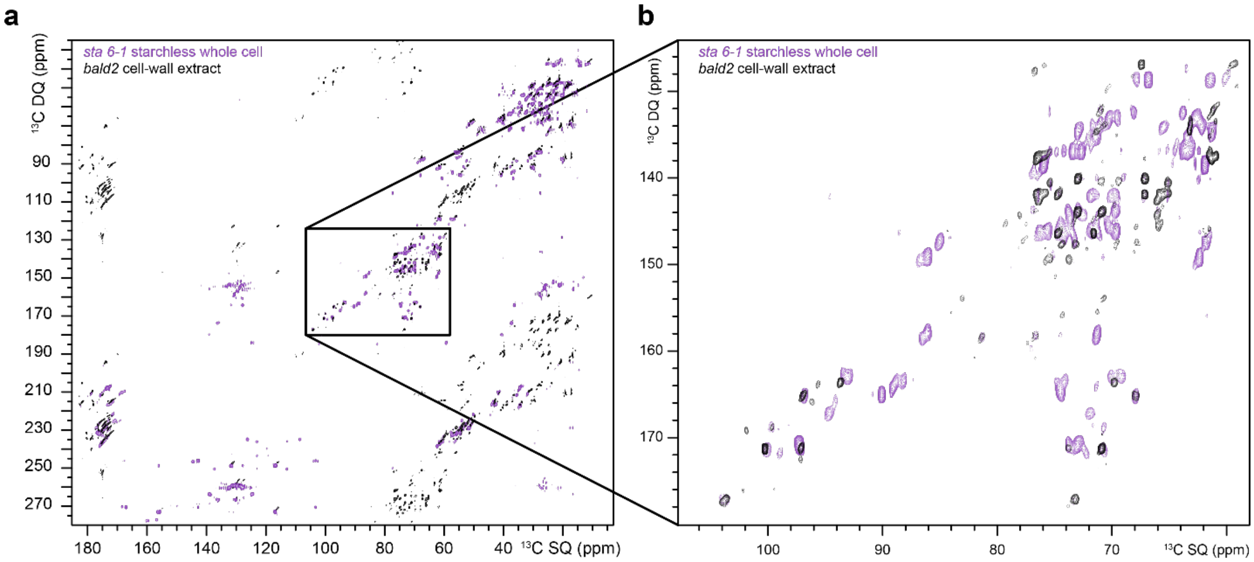

Supplementary Figure 2. Comparison of bald2 cell wall and stda 6-1 whole cell at 800 MHz. **a** Complete DP-INADEQUATE spectra of cell wall extracts (black) and whole cells (purple). **b** Zoom of the glycan region. At this temperature, 283 K, all glycans are detected quantitatively, and we know from GC-MS analysis that 40% of them are in the cell wall, but 60% are elsewhere in the cell (**Supplementary Table 8**). We therefore only expect at best a 40% overlap of the two spectra. Note that all peaks present in the cell wall spectra are indeed present in the whole cell spectra with the same chemical shifts.

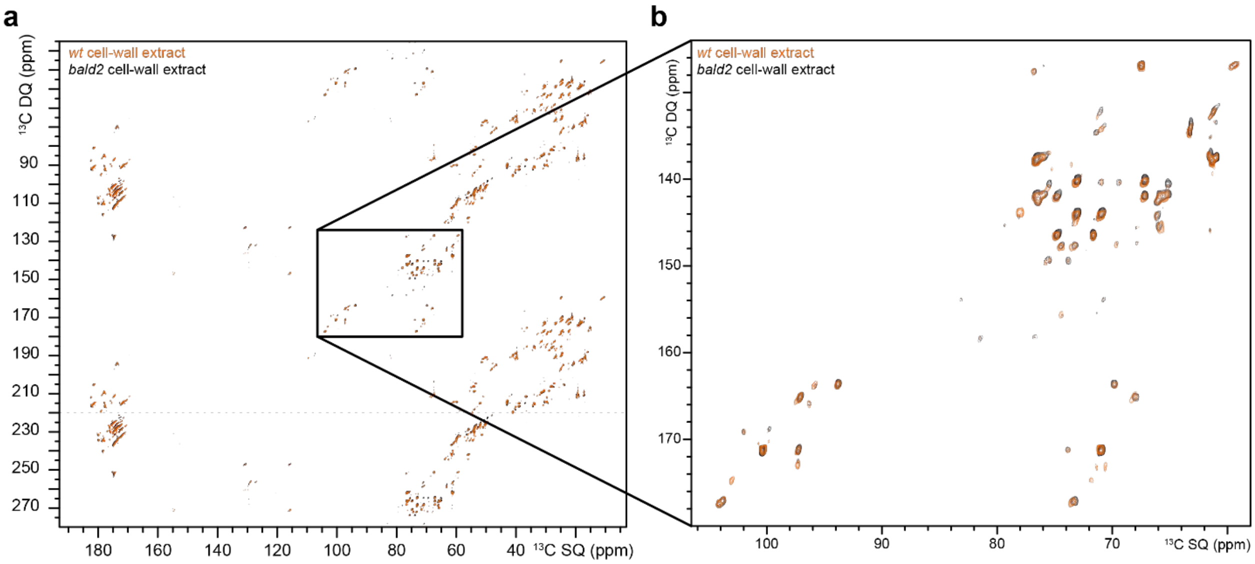

Supplementary Figure 3. Comparison of the cell wall spectra of *wt* and *bald2* strains. a Complete DP-INADEQUATE spectra of cell wall extracts from wild-type (orange) and flagella deficient (black) strains. **b** Zoom of the glycan region showing again very similar spectra. Spectra were recorded at fields of 18.8 T at 800 MHz, at 283 K.

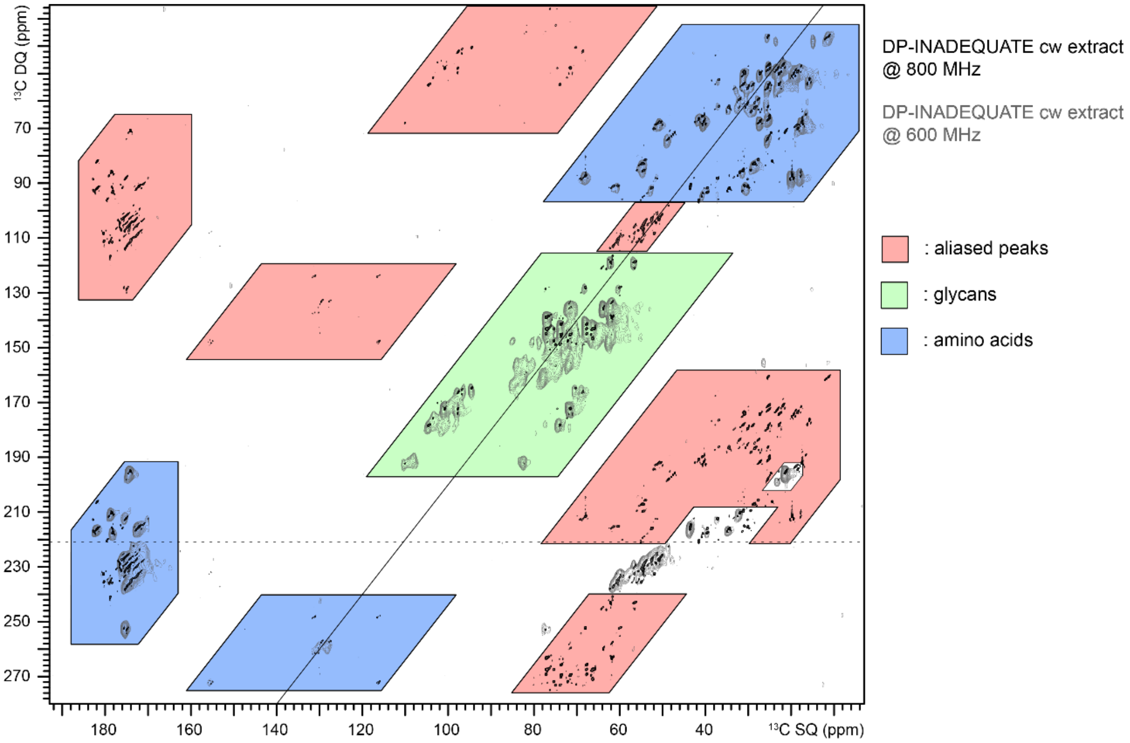

Supplementary Figure 4. Complete DP-INADEQUATE spectra of *C. reinhardtii's* cell wall. Comparison of spectra at two field strengths allow to assess resolution differences. Red regions are aliased peaks due to small indirect dimension used to save experimental time, but this artifact still allows assignment of glycans (green region used for assignment and quantification plotted on **Fig 2d**) and amino acids (blue region used for assignment and quantification reported in **Fig. 3a** and **Supplementary Fig. 5-7**).

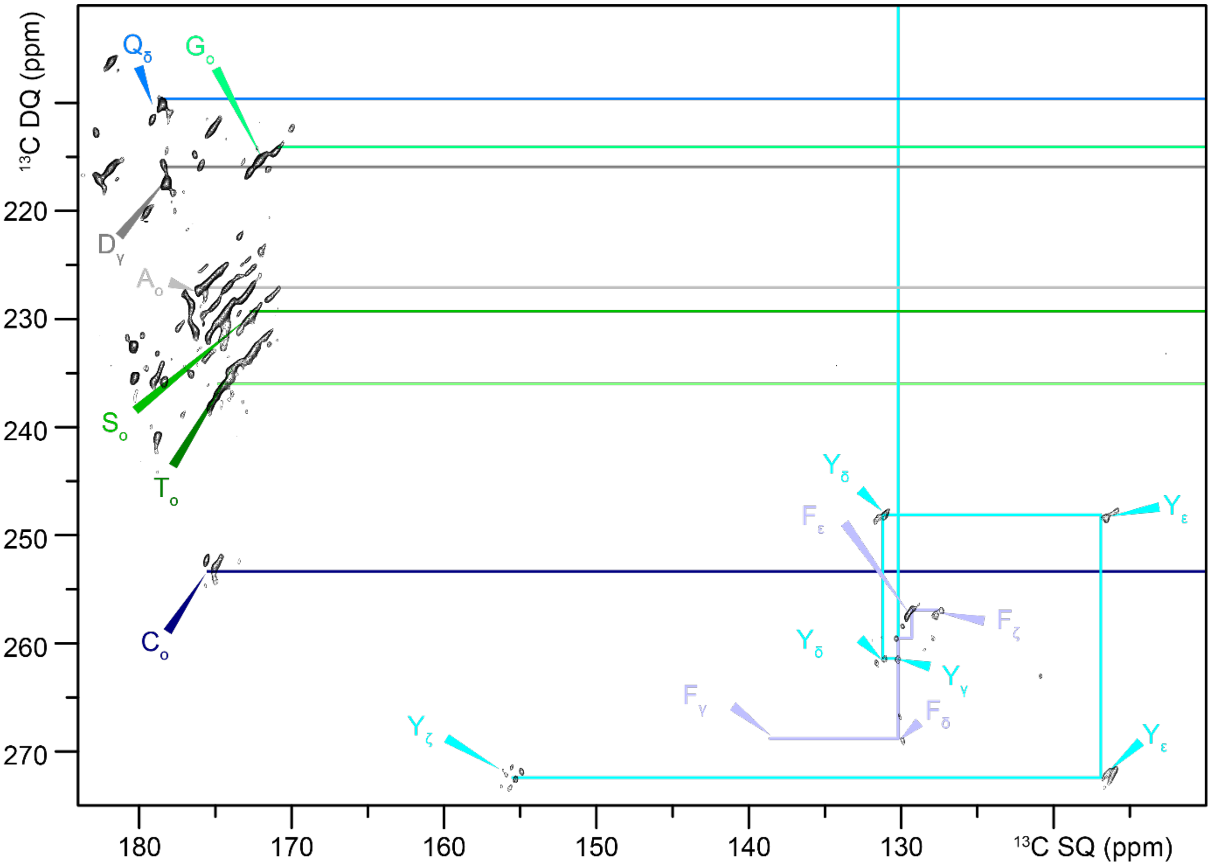

Supplementary Figure 5. Protein carbonyl assignment of *C. reinhardtii's* cell wall. Zoomed regions of the ^{13}C refocused DP-based *J*-INADEQUATE correlation spectrum with a short 3 s recycle delay. The zoom here shows the carbonyl and Tyr/Phe side chains correlations. This spectrum and the spectra plotted below on **Supplementary Figure 6** and **7** allow assessment of the overall resolution and provide examples of correlation paths for different amino acids (one-letter coded). For the overall ^{13}C-^{13}C *J*-INADEQUATE spectrum, see **Supplementary Fig. 4**.

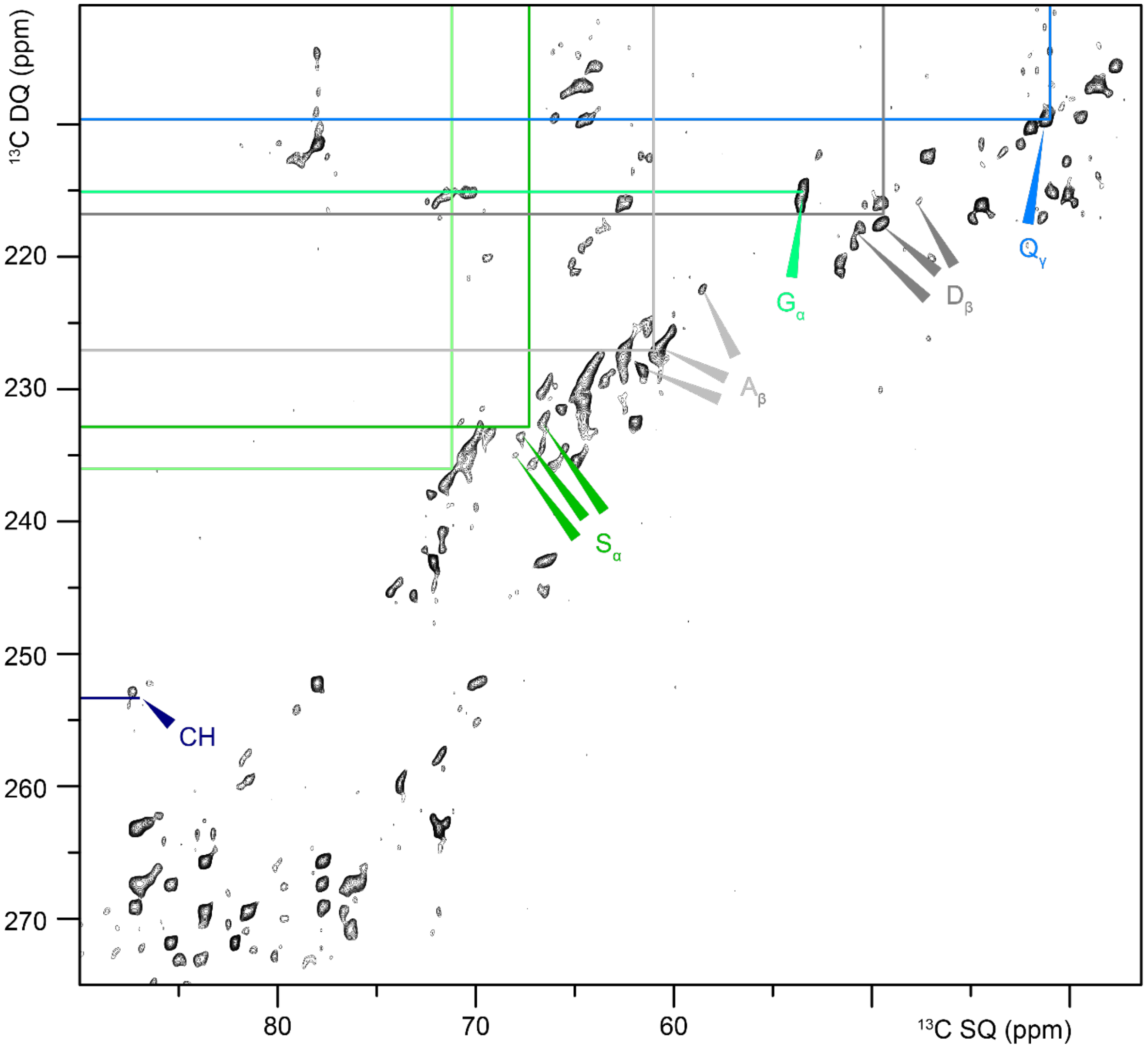

Supplementary Figure 6. Protein Cα assignment of *C. reinhardtii's* cell wall. Zoomed regions of the ^{13}C refocused DP-based *J*-INADEQUATE correlation spectrum with a short 3 s recycle delay. The zoom here shows the Cα of amino acid side chains. For the overall ^{13}C-^{13}C *J*-INADEQUATE spectrum, see **Supplementary Fig. 4**.

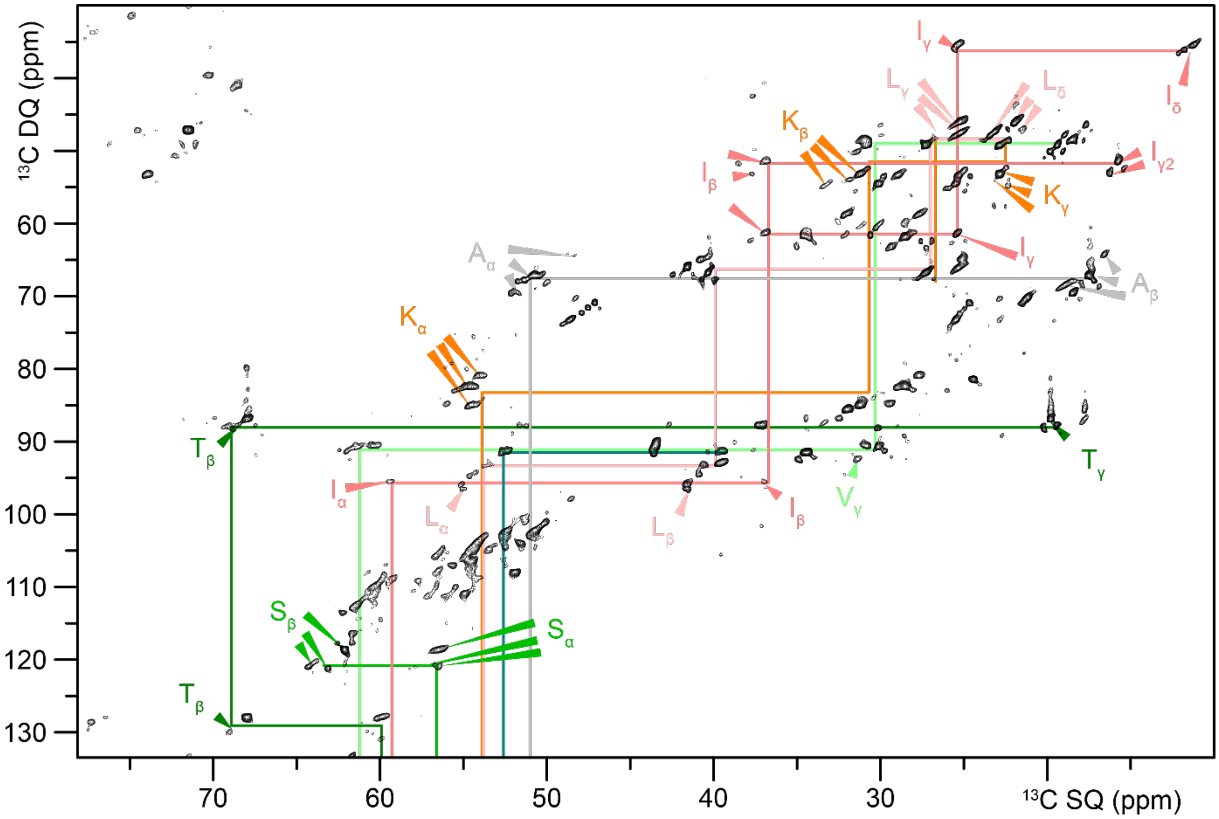

Supplementary Figure 7. Protein carbonyl assignment of *C. reinhardtii's* cell wall. Zoomed regions of the ^{13}C refocused DP-based *J*-INADEQUATE correlation spectrum with a short 3 s recycle delay. The zoom here shows the amino acid side chains correlations with one letter coding. For the overall ^{13}C-^{13}C *J*-INADEQUATE spectrum, see **Supplementary Fig. 4**.

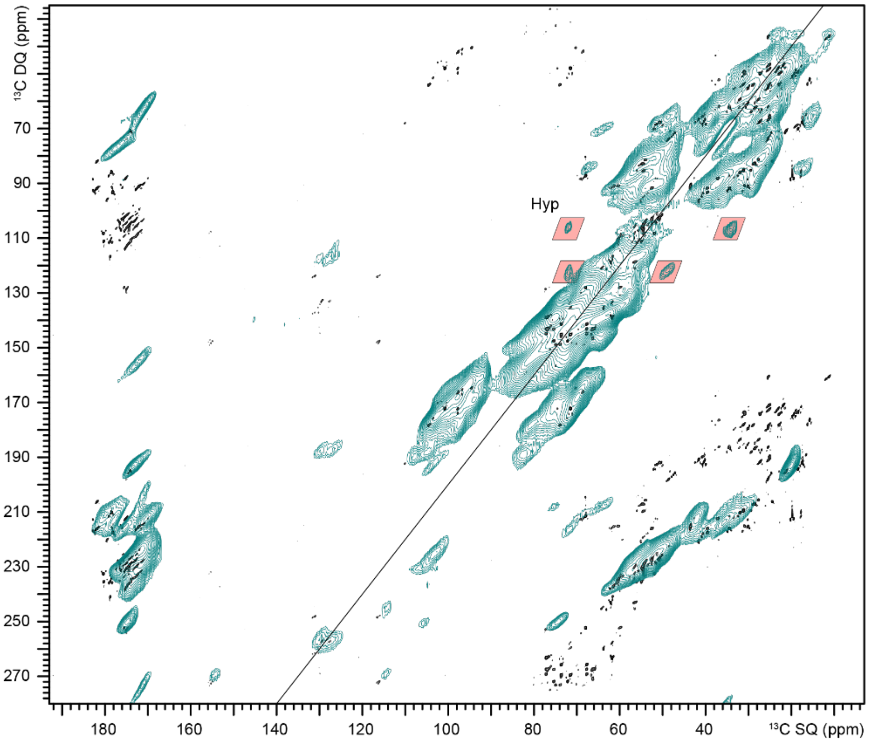

Supplementary Figure 8. ssNMR of *C. reinhardtii* cell wall at cryogenic and 283 K. Comparison of ^{13}C SQ-DQ *J*-INADEQUATE spectra recorded at 600 MHz using MAS-DNP and cryogenic temperature (100 K) (teal) and at 800 MHz using ssNMR at 283 K (black).

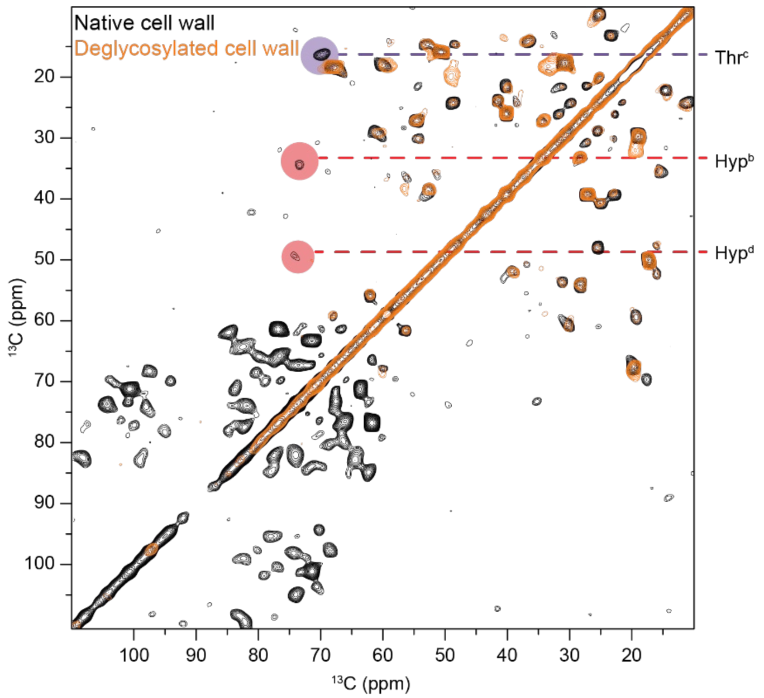

Supplementary Figure 9. Effect of deglycosylation on *C. reinhardtii* cell-wall extract. ^{13}C-^{13}C SQ-SQ DARR spectra with a mixing time of 50 ms of extracted cell wall (black) and deglycosylated extracted cell wall (orange). Resonances of O-glycosylated hydroxyproline and threonine are highlighted in red and purple, respectively.

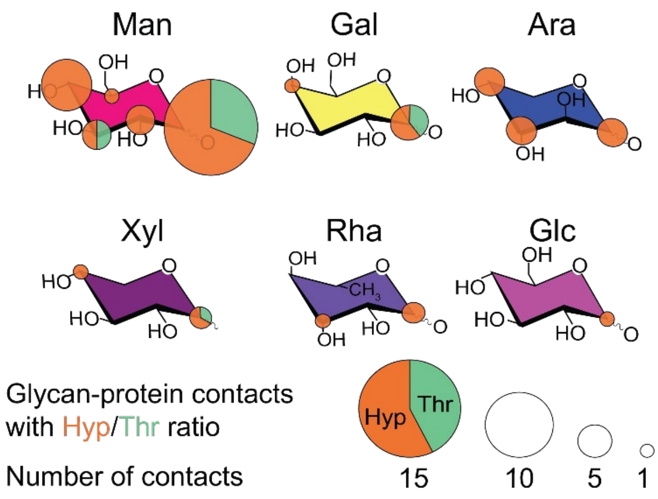

Supplementary Figure 10. Map of the resolved glycan-Hyp or -Thr spatial contacts. These contacts were detected by MAS-DNP. Pie chart diameters correspond to the number of contacts identified with a given glycan carbon. Sector area illustrates the propensity for this carbon to preferentially interact with Hyp (orange) or Thr (green).

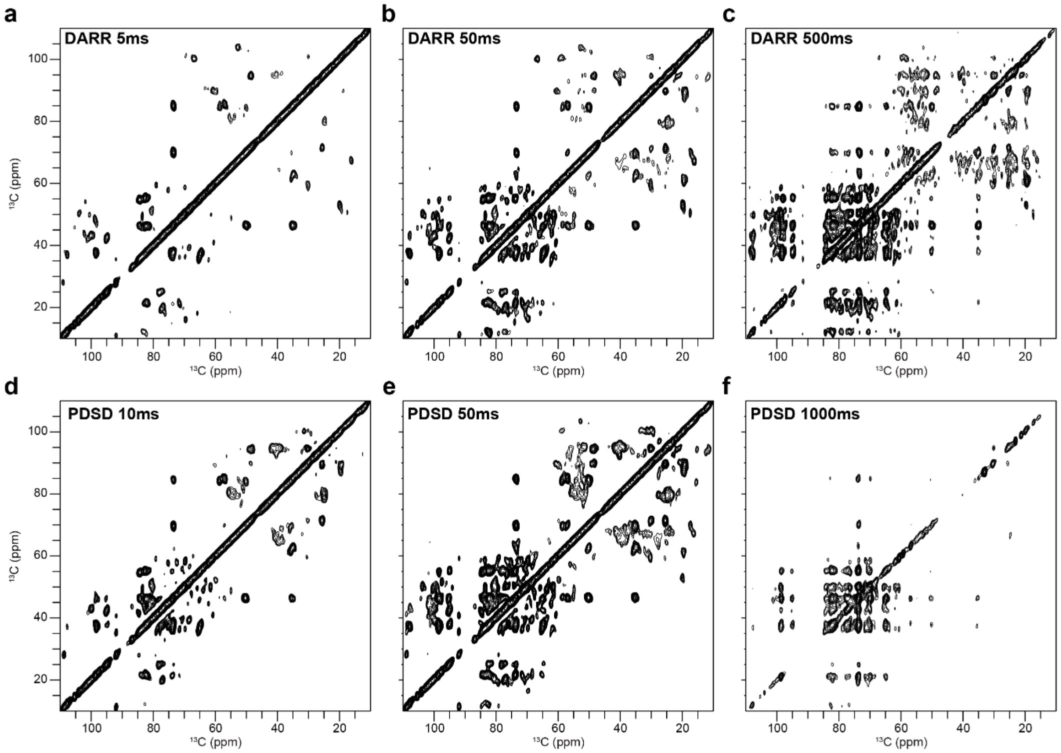

Supplementary Figure 11. DARR/PDSD build-up spectra. a-c DARR build-up with 5, 50 and 500 ms mixing times. **d-f** PDSD build-up with 10, 50 and 1000 ms mixing time.

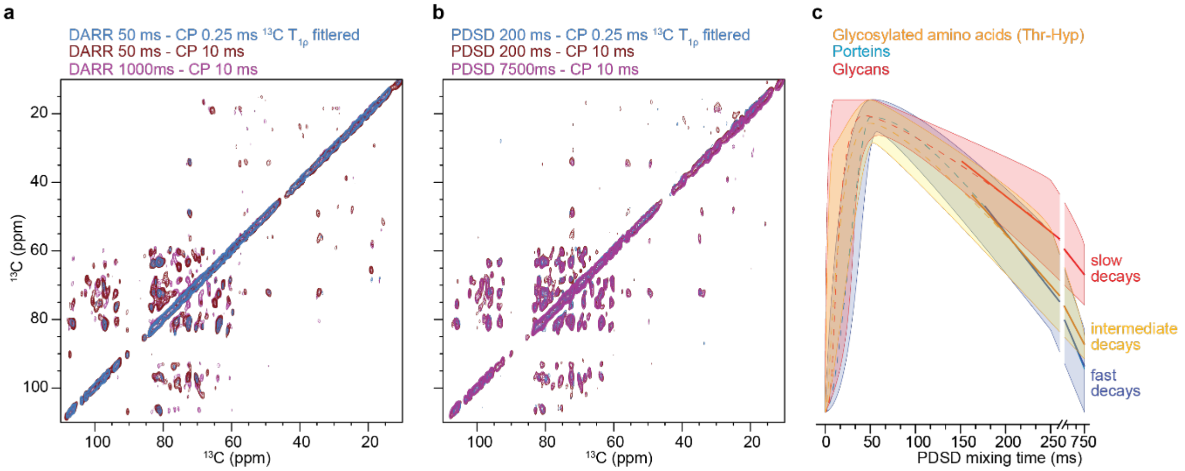

Supplementary Figure 12. DARR/PDSD 2D spectral editing. To understand the effect of different relaxations on spectral edition, we first applied long CP for both PDSD and DARR (**a** and **b** respectively, red spectra). Then by combining long CP and long DARR/PDSD mixing times (**a** and **b** respectively, purple spectra), we were able to further filter out the amino acid fraction. Finally, we show that the most efficient filter is a dedicated ^{13}C $T_{1\rho}$ filter (a and b respectively, blue spectra). All spectra were recorded with a 600 MHz spectrometer. **c** The PDSD cross peak normalized intensity build-ups show differences between glycans (red) and amino acids (blue), and the intermediate behavior of glycosylated amino acids (orange).

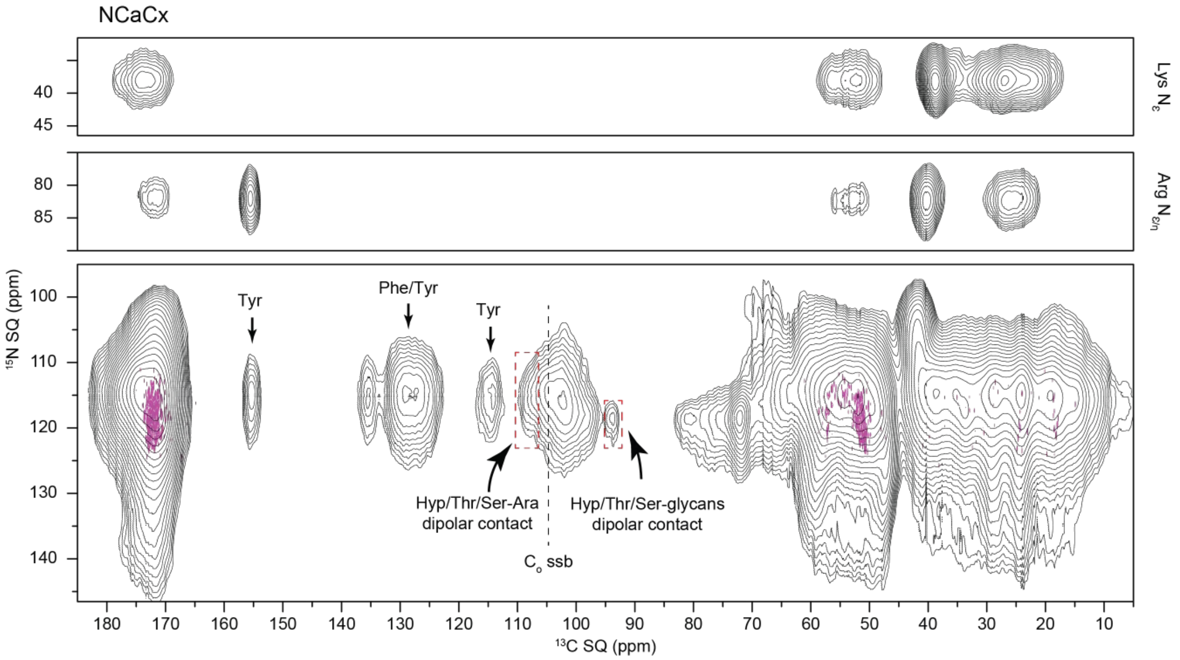

Supplementary Figure 13. NCaCx MAS-DNP and higher field spectra. NCaCx spectra of cell-wall extracts detected using MAS-DNP at 600 MHz, 100 K and 8kHz (black) and ssNMR at 800 MHz, 283 K and 13.5 kHz (purple). Allowing magnetisation to transfer from Cα to surrounding carbons, new peaks are detected. For example, O-glycosylated amino acids (hydroxyproline, threonine and serine) have their backbone nitrogen at ~115 and ~120 ppm correlating with anomeric carbons of glycans at ~110 ppm and ~95 ppm, respectively. Unfortunately, at 600 MHz and 8kHz spinning rate, previously NCa detected asparagine-glycan C_1 contacts (**Fig. 4d**) are now overlapping with the carbonyl spinning sideband (C_O ssb).

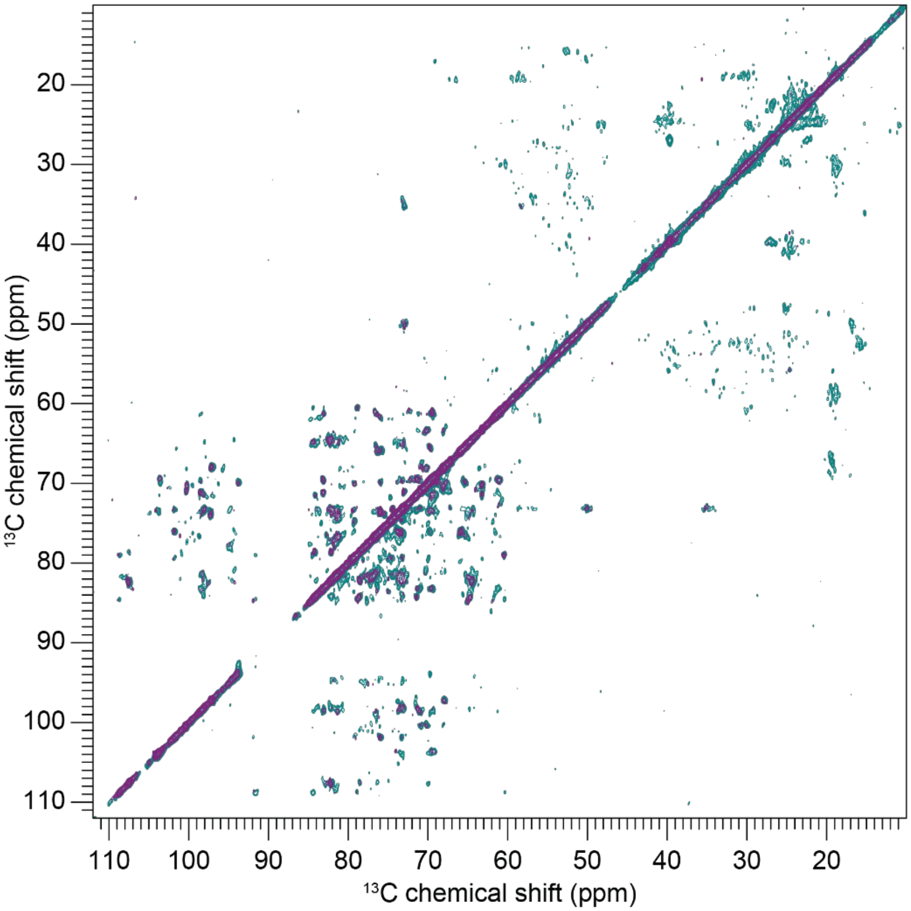

Supplementary Figure 14. 2D ^{13}C-^{13}C DARR water edition applied to *C. reinhardtii* cell wall. Control (teal) and water-edited (purple) 50 ms DARR spectra showing glycans and glycosylated amino acids to be more hydrated than other protein segments. Spectra were recorded on a 800 MHz spectrometer.

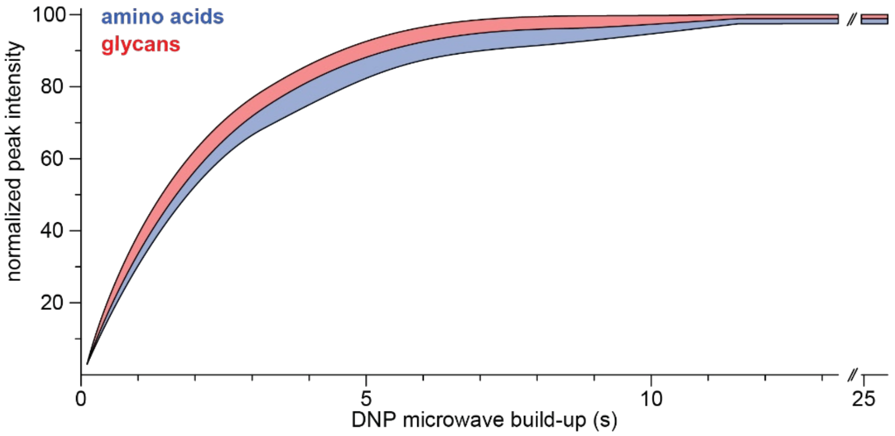

Supplementary Figure 15. DNP build-up of *C. reinhardtii's* cell wall extract. A slightly faster build-up is observed for glycans as compared to amino acids.

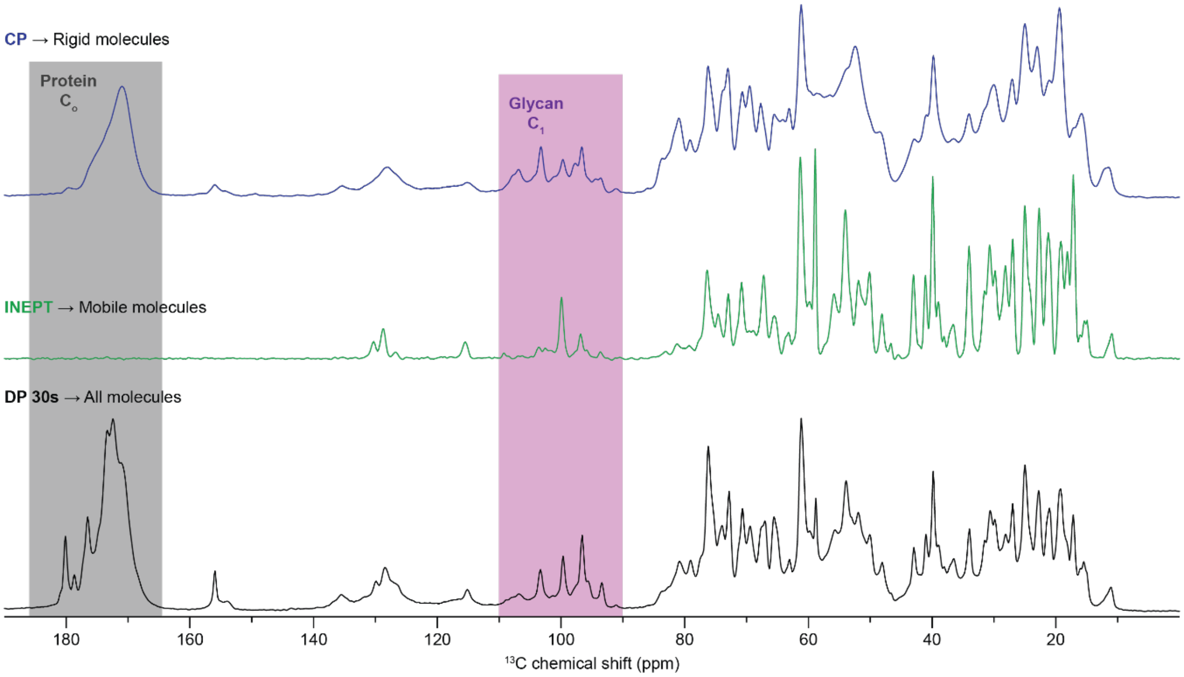

Supplementary Figure 16. 1D experiments revealing dynamics in *C. reinhardtii* cell wall. The CP spectrum (top, blue) selects rigid molecule segments, while the J-coupling-based refocused INEPT spectrum (middle, green) shows signals of highly mobile molecular segments when compared to the quantitative spectrum (bottom, black).

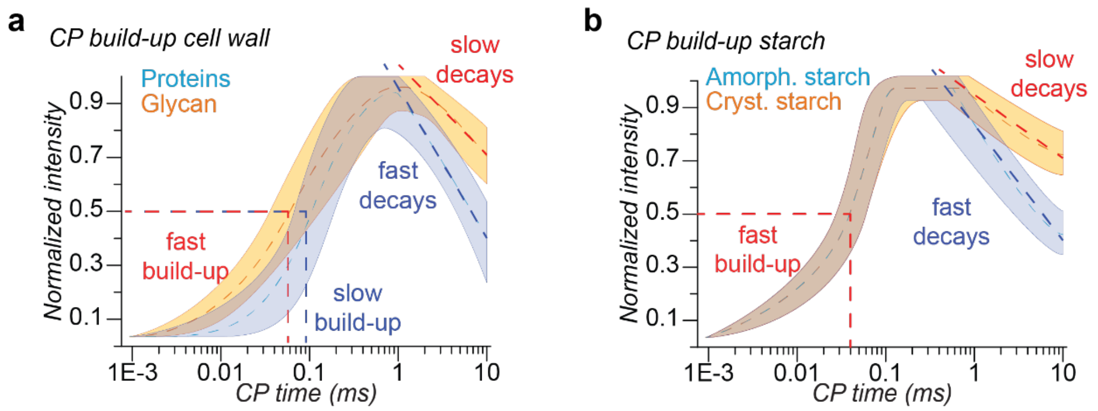

Supplementary Figure 17. CP build-up in *C. reinhardtii's* constituents. a CP build-up of amino acid and glycan carbons in the cell wall. **b** CP build-up of amorphous and crystalline starch carbons [15,16]. Details of the CP build-up for the cell wall and starch are given in **Supplementary Tables 17** and **18**, respectively. Fewer peaks were monitored compared to T_1 measurements, due to the lower resolution of the CP (**Supplementary Fig. 16**).

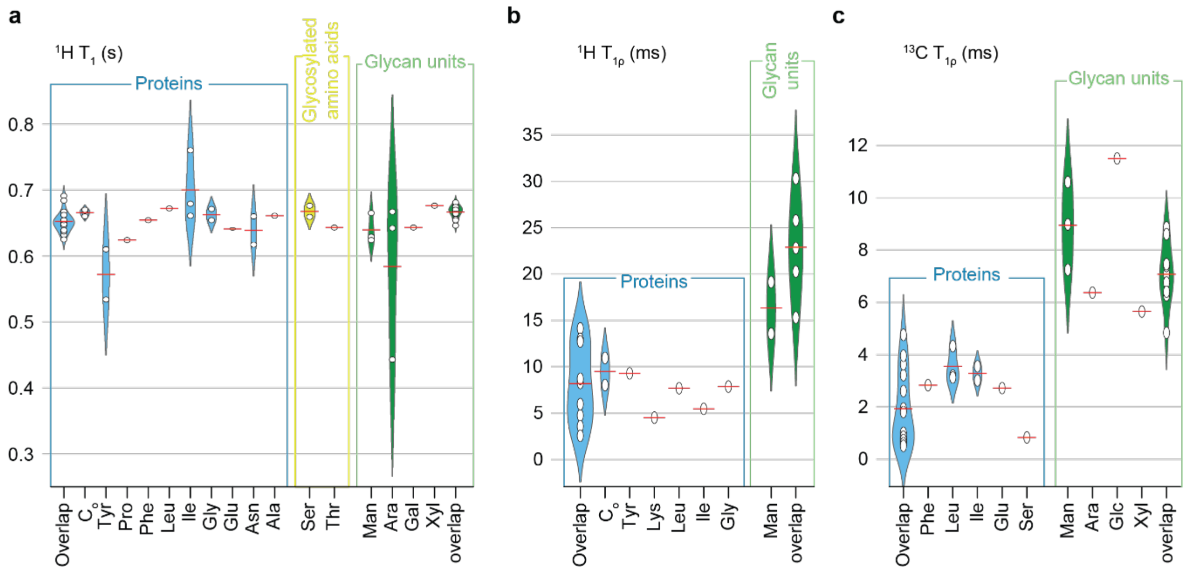

Supplementary Figure 18. Relaxation measurement of *C. reinhardtii's* cell wall. a ^{1}H spin-lattice (T_1) values of different amino acids and glycans. **b** ^{1}H and **c** ^{13}C $T_{1\rho}$ measurements of the same cell-wall extract. All fittings were performed on the most resolved peaks on the 1D spectra and separated based on the assignment obtained with the ^{13}C-^{13}C refocused *J*-INADEQUATE spectrum. Violin plots represent relaxation time dispersion within a single sample but corresponding to different protons/carbons from a specific residue or glycan unit.

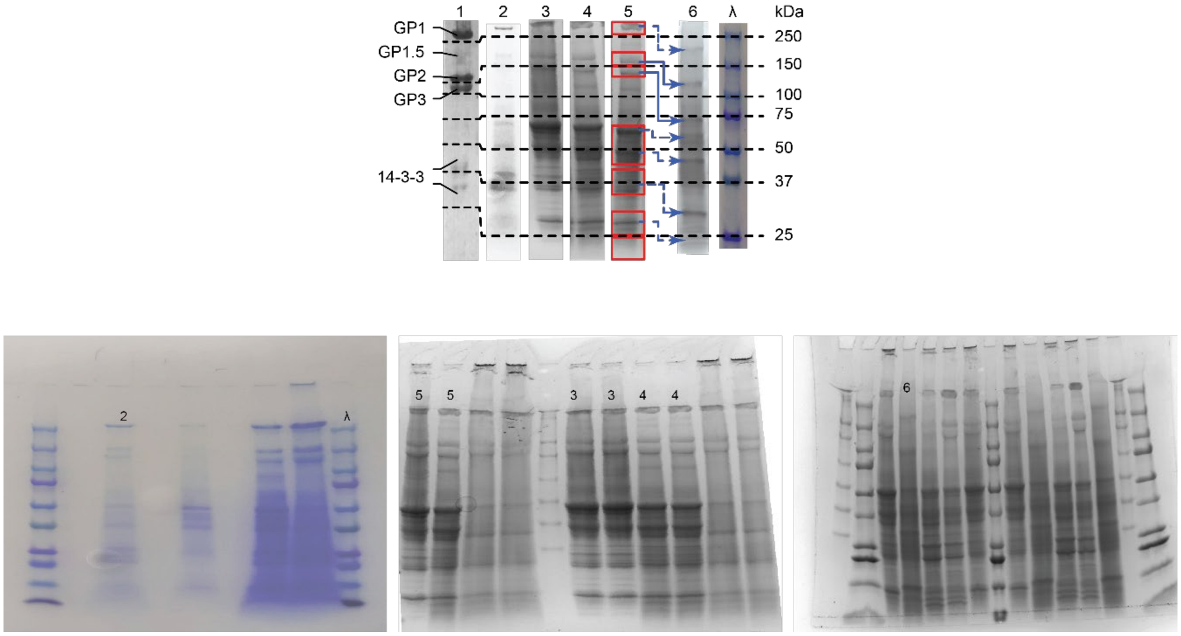

Supplementary Figure 19. Uncropped SD-PAGE gels. The uncropped gels were stained using Coomassie blue (0.05% w/v Coomassie Blue R-250) for 20-60 min, and destained with 30% v/v methanol/10% v/v glacial acetic acid for 5 h.

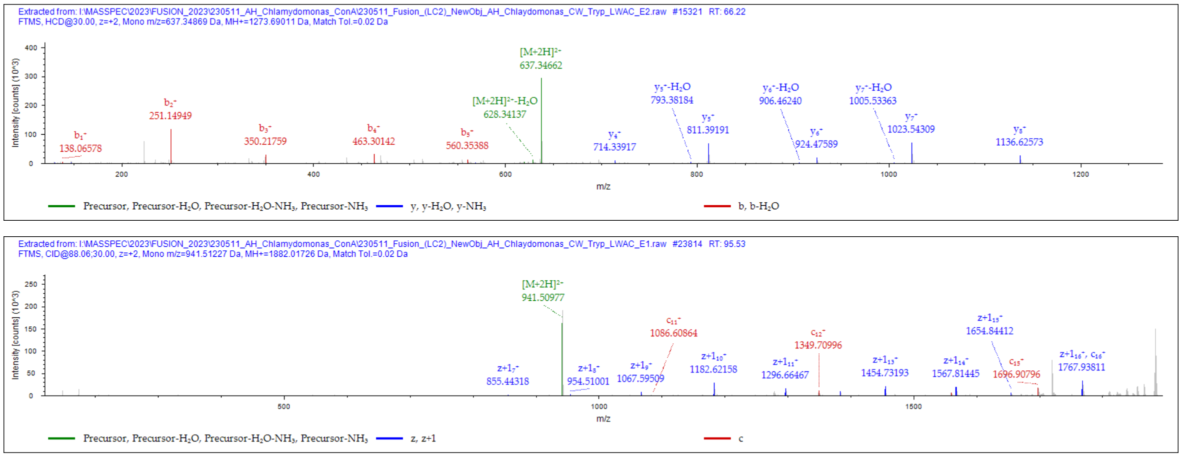

Supplementary Figure 20. Representative MS/MS spectra of glycopeptides. These hexose modified glycopeptides were identified in ConA-LWAC enriched fractions. Top spectrum is HCD fragmentation of HLVLPAATK (T8-Hex-Hex), z=+2, m/z=637.3487, derived from Protein kinase domain-containing protein. Bottom spectrum is ETciD fragmentation of VLSLASNDLVGTLPHAL (T12-Hex), z=+2, m/z=941.5123, derived from Guanylate cyclase domain-containing protein.

Supplementary Table 1. *wt* and *bald2* cell-wall glycan and amino acid composition. Glycan and amino acid composition of both strains are determined by GC/MS and HPLC, and compared with ssNMR, showing no significant differences. See **Supplementary Fig. 7** for details. Amino acids highlighted in orange are the amino acids that can be involved in O- or N-glycosylation.

	bald2		*wt*	
	MS	ssNMR	MS	ssNMR
**Man**	50.4	48.2	44.8	52.0
**Ara**	20.1	14.0	17.9	14.5
**Gal**	18.7	20.4	16.6	15.3
**Rha**	2.2	2.5	2.0	2.5
**Glc**	1.5	3.0	1.3	3.1
**Xyl**	1.5	2.2	1.3	2.5
**GlcNAc**	5.6	9.7	5.2	10.1

	bald2		*wt*	
	ssNMR	HPLC	ssNMR	HPLC
**Gln**	9.1	19.0 (0.3)	9.0	10.0
**Glu**	8.8		8.8	9.0
**Asn**	6.4	14.8 (0.3)	6.4	6.7
**Asp**	5.9		6.0	8.1
**Ala**	13.1	10.9 (0.3)	13.2	10.9
**Gly**	8.5	8.0 (0.3)	8.5	8.0
**Leu**	8.0	5.5 (0.3)	8.0	5.5
**Lys**	6.2	5.3 (0.1)	6.2	5.3
**Ser**	6.2	5.0 (0.2)	6.2	5.0
**Pro**	5.8	4.8 (0.2)	5.8	4.8
**Val**	5.7	5.6 (0.3)	5.6	5.6
**Arg**	5.1	3.5 (0.3)	5.1	3.5
**Thr**	4.3	4.7 (0.4)	4.3	4.6
**Met**	2.5	1.6 (0.2)	2.5	1.6
**Ile**	2.2	3.1 (0.3)	2.2	3.0
**Tyr**	1.5	2.4 (0.2)	1.5	2.4
**Phe**	0.7	2.8 (0.4)	0.7	2.8
**His**	*NA*	2.0 (0.1)	0.0	2.0
**Hyp**	*NA*	1.0 (0.1)	0.0	1.0

Supplementary Table 2. Glycosyl linkages determined by GC-MS in different samples.

Sample	*bald2* whole cell	*bald2* cw extracted	*bald2* cw extract	*wt* whole cell	*wt* cw extracted	*cw15* whole cell	*cw15* cw extracted	*Sta 6-1* whole cell	*Sta 6-1* cw extracted
t-Manp	2.8	2.6	7.2	2.0	1.3	2.6	21.1	11.5	10.8
2-Manp	**10.4**	**17.4**	**15.8**	**8.4**	**10.7**	6.0	1.5	**28.1**	**30.8**
3-Manp	0.0	0.0	0.0	0.0	2.0	2.1	3.4	1.5	0.0
2,3-Manp	3.2	3.1	5.4	3.9	1.6	2.3	**12.7**	8.1	8.5
2,4-Manp	0.0	0.0	0.9	2.2	1.3	0.0	0.0	0.0	0.0
t-Araf	1.4	1.4	1.7	1.0	1.1	**25.1**	7.7	4.0	2.5
t-Arap	0.8	1.5	1.4	0.0	1.0	0.0	3.8	3.7	2.1
2-Araf	2.6	4.0	6.4	1.9	2.3	4.3	**12.5**	8.6	8.5
2-Arap	0.0	0.0	0.0	0.0	0.0	0.0	1.7	1.5	2.5
2,4-Arap/2,5-Araf	0.0	0.0	0.0	0.0	0.0	0.0	2.0	4.1	3.1
t-Galp	3.8	8.5	5.0	3.5	4.6	2.8	3.4	7.6	7.2
t-Galf	1.5	2.4	3.1	2.5	1.6	1.5	5.7	4.9	4.5
4-Galp	0.0	0.0	0.0	0.0	0.0	0.0	2.3	1.1	1.5
3,4-Galp	0.0	0.0	0.0	0.0	0.0	2.9	5.3	0.0	0.0
t-Glcp	**17.6**	**12.2**	**15.3**	**19.8**	**14.0**	**18.7**	**13.6**	**8.9**	**13.2**
3-Glcp	0.9	0.2	2.5	0.7	0.6	1.7	1.5	1.6	1.7
4-Glcp	**42.7**	**33.9**	**20.2**	**42.0**	**42.0**	**18.1**	0.0	0.1	0.1
4,6-Glcp	3.3	2.9	2.0	4.5	4.6	1.9	0.5	1.0	1.4
3,4-Glcp	5.5	7.3	5.4	7.8	7.7	5.3	0.0	0.0	0.0
2,4-Rhap	0.0	0.0	2.9	0.0	0.0	0.0	1.3	1.0	1.6
3-Hexf	3.5	2.7	4.8	0.0	3.5	4.8	0.1	2.7	0.0

Supplementary Table 3. Full ^{13}C ssNMR glycan assignment of *C. reinhardtii bald2*'s cell wall.

Sugar Type	C1	C2	C3	C4	C5	C6	Ac	Met
**Mannose units**								
Man1	104.5	73.9	74.9	81.7	77.2	61.8		
Man2	97.7	74.6	76.2	65.7	77.2	61.4		
Man3	100.8	71.5	73.6	67.8	77.1	61.8		
Man4	103.6	72.2	75.4	67.9	77.2	61.9		
Man5	100.2	76.8	71.4	68.4	77.7	65.7		
Man6	102.4	67.8	77.2	65.7	76.0	62.1		
Man7	101.7	67.9	77.1	65.6	76.0	62.1		
Man8	103	71.1	77.1	67.8	75.3	63.1		
Man9	94.6	69.9	78.1	68.3	74.1	64.9		
Man10	96.7	70.4	76.8	65.8	76.0	62.2		
Man11	97.6	68.6	80	66.2	76.9	61.9		
Man12	96.8	68.6	80.8	66.3	76.9	61.5		
Man13	94.5	70.2	78.6	66.7	77	61.8		
Man14	99.2	73.6	78.5	65.9	70.5	66.2		
**Arabinose units**								
Ara1	110.3	82.4	77.6	81.8	61.8			
Ara2	110.1	82.2	77.7	81.7	61.8			
Ara3	109.8	81.9	77.2	81.4	66.1			
Ara4	109.8	81.8	75	81.3	67.2			
**Galactose units**								
Gal1	103.5	72.1	75.4	67.7	72.2	61.8		
Gal2	98.5	72.4	73.7	71.7	75.1	62.9		
Gal3	105.8	73.3	75	81.1	74.8	64.8		
**Acetyl-methyl groups**								
Ac1							23.4	175.5
Ac2							22.6	175.2
Ac3							23.8	173.4
Ac4							21.5	175.6
**Rhamnose units**								
Rha1	100.4	77.1	70.2	71.7	70.3	17.8		
Rha2	100.4	77.1	70.2	71.9	69.8	17.6		
Rha3	100.3	70.2	78.7	73.6	70.9	17.4		
**Glucose units**								
Glc1	100.4	69.6	74.1	72.5	70.9	63.6		
Glc2	100	76.8	71.3	63.8	71.8	61.9		
**Xylose units**								
Xyl1	97.7	76.1	77.2	67.7	60.2			
Xyl2	100.3	70.8	75.9	74.8	64.9			
Xyl3	100.9	77.4	75.1	79.5	67.8			

Supplementary Table 4. ^{13}C ssNMR glycan assignment and comparison with literature values.

Glycan units		SQ	SQ		Reference 1		Reference 2	
Man1					*Kang 2018*[14]		*Gorin 1975*[17]	
	C1	104.5	73.9	C2	101	69.4	94.6	72.3
	C3	73.9	74.9	C2	69.4	76.2	72.3	74.1
	C4	74.9	81.7	C3	76.2	83.4	74.1	67.8
	C4	81.7	77.2	C5	83.4	77.1	67.8	77.2
	C5	77.2	61.8	C6	77.1	61.6	77.2	62.1
Man2					*Bradbury 1984*[18]		*Gorin 1975*[17]	
	C1	97.7	74.6	C2	95.2	72.8	94.6	72.3
	C3	74.6	76.2	C2	72.8	74.8	72.3	74.1
	C3	76.2	65.7	C4	74.8	68.3	74.1	67.8
	C5	65.7	77.2	C4	68.3	77.6	67.8	77.2
	C5	77.2	61.4	C6	77.6	62.6	77.2	62.1
Man3					*Bradbury 1984*[18]		*Lundborg 2011*[19]	
	C1	100.8	71.5	C2	95.2	72.8	95	71.1
	C3	71.5	73.6	C2	72.8	74.8	71.1	79
	C3	73.6	67.8	C4	74.8	68.3	79	66.6
	C5	67.8	77.1	C4	68.3	77.6	66.6	71.8
	C5	77.1	61.8	C6	77.6	62.6	71.8	66.4
Man4					*Gorin 1975*[17]			
	C1	103.6	72.2	C2	94.6	72.3		
	C3	72.2	75.4	C2	72.3	74.1		
	C3	75.4	67.9	C4	74.1	67.8		
	C5	67.9	77.2	C4	67.8	77.2		
	C5	77.2	61.9	C6	77.2	62.1		
Man5					*Lundborg 2011*[19]		*Gorin 1975*[17]	
	C1	100.2	76.8	C2	97.8	77.9	94.6	72.3
	C2	76.8	71.4	C3	77.9	70.7	72.3	74.1
	C3	71.4	68.4	C4	70.7	68.3	74.1	67.8
	C5	68.4	77.7	C6	68.3	73.9	67.8	77.2
	C5	77.7	65.7	C4	73.9	62.4	77.2	62.1
Man6					*Gorin 1975*[17]		*Lundborg 2011*[19]	
	C1	102.4	67.8	C2	94.6	72.3	95	71.1
	C3	67.8	77.2	C2	72.3	74.1	71.1	79
	C3	77.2	65.7	C4	74.1	67.8	79	66.6
	C5	65.7	76.0	C4	67.8	77.2	66.6	71.8
	C5	76.0	62.1	C6	77.2	62.1	71.8	66.4
Man7					*Gorin 1975*[17]		*Lundborg 2011*[19]	
	C1	101.7	67.9	C2	94.6	72.3	95	71.1
	C3	67.9	77.1	C2	72.3	74.1	71.1	79
	C3	77.1	65.6	C4	74.1	67.8	79	66.6
	C5	65.6	76.0	C4	67.8	77.2	66.6	71.8
	C5	76.0	62.1	C6	77.2	62.1	71.8	66.4
Man8					*Gorin 1975*[17]		*Lundborg 2011*[19]	
	C1	103	71.1	C2	94.6	72.3	95	71.1
	C3	71.1	77.1	C2	72.3	74.1	71.1	79
	C3	77.1	67.8	C4	74.1	67.8	79	66.6
	C5	67.8	75.3	C4	67.8	77.2	66.6	71.8
	C5	75.3	63.1	C6	77.2	62.1	71.8	66.4
Man9					*Gorin 1975*[17]		*Lundborg 2011*[19]	
	C1	94.6	69.9	C2	94.6	72.3	95	71.1

	C3	69.9	78.1	C2	72.3	74.1	71.1	79
	C3	78.1	68.3	C4	74.1	67.8	79	66.6
	C5	68.3	74.1	C4	67.8	77.2	66.6	71.8
	C5	74.1	64.9	C6	77.2	62.1	71.8	66.4
Man10					*Gorin 1975*[17]		*Lundborg 2011*[19]	
	C1	96.7	70.4	C2	94.6	72.3	95	71.1
	C3	70.4	76.8	C2	72.3	74.1	71.1	79
	C3	76.8	65.8	C4	74.1	67.8	79	66.6
	C5	65.8	76.0	C4	67.8	77.2	66.6	71.8
	C5	76.0	62.2	C6	77.2	62.1	71.8	66.4
Man11					*Gorin 1975*[17]		*Lundborg 2011*[19]	
	C1	97.6	68.6	C2	94.6	72.3	95	71.1
	C3	68.6	80	C2	72.3	74.1	71.1	79
	C3	80	66.2	C4	74.1	67.8	79	66.6
	C5	66.2	76.9	C4	67.8	77.2	66.6	71.8
	C5	76.9	61.9	C6	77.2	62.1	71.8	66.4
Man12					*Gorin 1975*[17]		*Lundborg 2011*[19]	
	C1	96.8	68.6	C2	94.6	72.3	95	71.1
	C3	68.6	80.8	C2	72.3	74.1	71.1	79
	C3	80.8	66.3	C4	74.1	67.8	79	66.6
	C5	66.3	76.9	C4	67.8	77.2	66.6	71.8
	C5	76.9	61.5	C6	77.2	62.1	71.8	66.4
Man13					*Gorin 1975*[17]		*Lundborg 2011*[19]	
	C1	94.5	70.2	C2	94.6	72.3	95	71.1
	C3	70.2	78.6	C2	72.3	74.1	71.1	79
	C3	78.6	66.7	C4	74.1	67.8	79	66.6
	C5	66.7	77	C4	67.8	77.2	66.6	71.8
	C5	77	61.8	C6	77.2	62.1	71.8	66.4
Man14					*Lunborg 2011*[19]			
	C1	99.2	73.6	C2	94.9	71.2		
	C3	73.6	78.5	C2	71.2	79.3		
	C3	78.5	65.9	C4	79.3	66.9		
	C5	65.9	70.5	C6	66.9	71.9		
	C5	70.5	66.2	C4	71.9	66.8		
Ara1					*wang 2014*[20]			
	C1	110.3	82.4	C2	110	82.2		
	C2	82.4	77.6	C3	82.2	77.7		
	C4	77.6	81.8	C3	77.7	84.9		
	C4	81.8	61.8	C5	84.9	62.3		
Ara2					*wang 2014*[21]			
	C1	110.1	82.2	C2	110	82.2		
	C2	82.2	77.7	C3	82.2	77.7		
	C4	77.7	81.7	C3	77.7	84.9		
	C4	81.7	61.8	C5	84.9	62.3		
Ara3					*wang 2014*[20]		*Phyo 2017a*[22]	
	C1	109.8	81.9	C2	110	82.2	108.4	81.8
	C2	81.9	77.2	C3	82.2	77.7	81.8	77.8
	C4	77.2	81.4	C3	77.7	84.9	77.8	83.1
	C4	81.4	66.1	C5	84.9	62.3	83.1	67.8
Ara4					*wang 2014*[20]		*Phyo 2017a*[22]	
	C1	109.8	81.8	C2	110	82.2	108.4	81.8
	C2	81.8	75	C3	82.2	77.7	81.8	77.8
	C4	75	81.3	C3	77.7	84.9	77.8	83.1
	C4	81.3	67.2	C5	84.9	62.3	83.1	67.8

Gal1					*Wang 2014*[20]		
	C1	103.5	72.1	C2	103.4	74	
	C3	72.1	75.4	C2	74	76.4	
	C3	75.4	67.7	C4	76.4	73.8	
	C5	67.7	72.2	C4	73.8	74.8	
	C5	72.2	61.8	C6	74.8	61.7	
Gal2					*Arnold 2018*[15]		
	C1	98.5	72.4	C2	100.1	73.4	
	C3	72.4	73.7	C2	73.4	74.9	
	C3	73.7	71.7	C4	74.9	74.9	
	C5	71.7	75.1	C4	74.9	71.7	
	C5	75.1	62.9	C6	71.7	54.3	
Gal3					*Rondeau-Mouro 2008*[23]		
	C1	105.8	73.3	C2	105.5	72.9	
	C3	73.3	75	C2	72.9	74.4	
	C4	75	81.1	C3	74.4	78.8	
	C4	81.1	74.8	C5	78.8	75.7	
	C5	74.6	64.9	C6	75.7	61.6	
Ac1					Kang 2018[14]		King 2017[24]
	Ac	175.5	23.4	Met			
Ac2							
	Ac	175.2	22.6	Met			
Ac3							
	Ac	173.4	23.8	Met			
Ac4							
	Ac	175.6	21.5	Met			
Rha1					*Habibi 2004*[25]		
	C1	100.4	77.1	C2	99.2	77.5	
	C2	77.1	70.2	C3	77.5	70.2	
	C4	70.2	71.7	C3	70.2	71.3	
	C4	71.7	70.3	C5	71.3	69.8	
	C5	70.3	17.8	C6	69.8	17.5	
Rha2					*Habibi 2004*[25]		
	C1	100.4	77.1	C2	99.2	77.5	
	C2	77.1	70.2	C3	77.5	70.2	
	C4	70.2	71.9	C3	70.2	71.3	
	C4	71.9	69.8	C5	71.3	69.8	
	C5	69.8	17.6	C6	69.8	17.5	
Rha3					*Laguri 2018*[26]		
	C1	100.3	70.2	C2	101.2	71.2	
	C3	70.2	78.7	C2	71.2	81.2	
	C3	78.7	73.6	C4	81.2	73.8	
	C4	73.6	70.9	C5	73.8	70.2	
	C5	70.9	17.4	C6	70.2	18.1	
Glc1					*Pfeffer 1984*[27]		
	C1	100.4	69.6	C2	92.9	70.5	
	C3	69.6	74.1	C2	70.5	73	
	C3	74.1	72.5	C4	73	72.6	
	C4	72.5	70.9	C5	72.6	71.6	
	C5	70.9	63.6	C6	71.6	63.7	
Glc2					*Poulhazan 2018*[15]		
	C1	100	76.8	C2	100.5	72.3	
	C2	76.8	71.3	C3	72.3	73.7	

	C3	71.3	63.8	C4	73.7	70.4		
	C5	63.8	71.8	C4	70.4	73.7		
	C5	71.8	61.9	C6	73.7	61.1		
Xyl1					*Gorin 1975*[17]		*Gorin 1975*[17]	
	C1	97.7	76.1	C2	100.6	72.3	97.6	75.1
	C3	76.1	77.2	C2	72.3	74.3	75.1	76.9
	C3	77.2	67.7	C4	74.3	70.4	76.9	70.3
	C4	67.7	60.2	C5	70.4	62	70.3	66.3
Xyl2					*Wang 2014*[20]			
	C1	100.3	70.8	C2	102.4	73.9		
	C3	70.8	75.9	C4	73.9	76.7		
	C3	75.9	74.8	C2	76.7	74.6		
	C4	74.8	64.9	C5	74.6	63.6		
Xyl3					*Wang 2014*[20]			
	C1	100.9	77.4	C2	102.2	77.6		
	C2	77.4	75.1	C3	77.6	73.7		
	C4	75.1	79.5	C3	73.7	77.4		
	C4	79.5	67.8	C5	77.4	63.8		
Unk 1								
	C1	102.4	71.8	C2				
	C2	71.8	69.8	C3				
	C4	75.9	69.8	C3				
	C4	75.9	62.1	C5				
Unk 2								
	C1	100.2	76.9	C2				
	C2	76.9	71.4	C3				
	C4	83.7	71.4	C3				
	C4	83.7	61.7	C5				

Supplementary Table 5. Glycan integrals used for ssNMR quantification. Values in red correspond to peaks overlapping with other types of glycans, and therefore cannot be used for quantification. Nevertheless, as explained before[16], overlapping peaks among same glycan types, in orange, can be used for quantification.

Glycan unit	peak int.		SQ	SQ		peak int.	mono./poly.
Man 1	3.4E+07	C1	104.5	73.9	C2	1.9E+07	poly
	1.6E+07	C3	74.9	73.9	C2	1.5E+07	
	**3.0E+06**	C4	81.7	74.9	C3	**9.9E+06**	
	**8.1E+06**	C4	81.7	77.2	C5	**7.3E+06**	
	8.6E+07	C5	77.0	61.8	C6	**1.2E+07**	
Man 2	1.6E+07	C1	97.7	74.6	C2	6.6E+06	poly
	**6.0E+06**	C3	76.2	74.6	C2	**7.0E+06**	
	7.9E+06	C3	76.2	65.7	C4	**4.3E+06**	
	5.5E+07	C5	77.2	65.7	C4	**6.3E+07**	
	8.6E+07	C5	76.9	61.5	C6	**1.7E+07**	
Man 3	8.1E+07	C1	100.8	71.5	C2	6.3E+07	poly
	**4.4E+07**	C3	73.6	71.8	C2	**4.8E+07**	
	4.6E+07	C3	73.6	67.8	C4	**4.2E+07**	
	2.4E+07	C5	77.1	67.8	C4	**2.2E+07**	
	1.3E+06	C5	77.7	59.6	C6	1.4E+06	
Man 4	**1.0E+07**	C1	103.5	72.3	C2	**6.2E+06**	poly
	**2.8E+07**	C3	75.4	72.2	C2	**3.7E+07**	
	**1.8E+07**	C3	75.4	67.7	C4	**2.2E+07**	
	2.4E+07	C5	77.2	67.7	C4	**2.2E+07**	
	8.6E+07	C5	77.0	61.8	C6	**1.2E+07**	
Man 5	**2.8E+06**	C1	100.2	76.8	C2	**2.7E+06**	poly
	**5.1E+06**	C2	76.8	71.3	C3	**4.9E+06**	
	2.7E+06	C3	71.4	68.4	C4	**3.2E+06**	
	1.1E+06	C5	77.7	65.7	C6	1.8E+06	
	9.0E+05	C5	77.7	68.4	C4	**3.1E+06**	
Man 6	5.0E+06	C1	102.4	67.8	C2	2.2E+06	poly
	2.4E+07	C3	77.2	67.8	C2	**2.2E+07**	
	5.5E+07	C3	77.2	65.7	C4	**6.3E+07**	
	7.9E+06	C5	76.0	65.7	C4	**4.3E+06**	

	5.8E+06	C5	76.0	62.1	C6	**1.0E+07**	
Man 7	2.9E+06	C1	101.7	67.9	C2	3.0E+06	poly
	2.4E+07	C3	77.1	67.9	C2	**2.2E+07**	
	5.5E+07	C3	77.1	65.6	C4	**6.3E+07**	
	7.9E+06	C5	76.0	65.6	C4	**4.3E+06**	
	5.8E+06	C5	76.0	62.1	C6	**1.0E+07**	
Man 8	3.6E+06	C1	103.0	71.1	C2	7.0E+06	poly
	**5.1E+06**	C3	77.1	71.1	C2	**4.9E+06**	
	2.4E+07	C3	77.1	67.8	C4	**2.2E+07**	
	**1.8E+07**	C5	75.3	67.8	C4	**2.2E+07**	
	**2.6E+06**	C5	75.3	63.1	C6	2.3E+06	
Man 9	1.8E+07	C1	94.6	69.9	C2	**1.1E+07**	mono
	6.7E+06	C3	78.1	69.9	C2	**9.3E+06**	
	2.8E+06	C3	78.1	68.3	C4	**3.1E+06**	
	4.6E+07	C5	74.1	68.3	C4	**8.7E+05**	
	**4.4E+06**	C5	74.1	64.9	C6	**3.2E+06**	
Man 10	8.1E+06	C1	96.7	70.4	C2	4.3E+06	mono
	3.6E+06	C3	76.8	70.4	C2	3.6E+06	
	5.5E+07	C3	76.8	65.8	C4	**6.3E+07**	
	7.9E+06	C5	76.0	65.8	C4	**4.3E+06**	
	5.8E+06	C5	76.0	62.2	C6	**1.0E+07**	
Man 11	2.7E+07	C1	97.6	68.6	C2	**2.2E+07**	poly
	5.8E+06	C3	80.1	68.3	C2	6.2E+06	
	6.7E+06	C3	80.0	66.2	C4	2.4E+07	
	**5.5E+07**	C5	76.9	66.2	C4	**6.3E+07**	
	**8.6E+07**	C5	76.9	61.9	C6	**1.7E+07**	
Man 12	1.1E+07	C1	96.8	68.6	C2	**2.2E+07**	mono
	2.4E+06	C3	80.8	68.6	C2	3.2E+06	
	**3.5E+06**	C3	80.8	66.3	C4	4.9E+06	
	**5.5E+07**	C5	76.9	66.3	C4	**6.3E+07**	
	**8.6E+07**	C5	76.9	61.5	C6	**1.7E+07**	
Man 13	**1.8E+07**	C1	94.5	70.2	C2	**1.1E+07**	mono
	**6.7E+06**	C3	78.6	70.2	C2	**9.3E+06**	
	4.4E+06	C3	78.6	66.7	C4	8.2E+06	

	**5.5E+07**	C5	77.0	66.7	C4	**6.3E+07**	
	**8.6E+07**	C5	77.0	61.8	C6	**1.2E+07**	
Man 14	8.3E+05	C1	99.2	73.6	C2	4.2E+06	mono
	1.6E+06	C3	78.5	73.6	C2	1.8E+06	
	1.7E+06	C3	78.5	66.1	C4	3.5E+06	
	**2.6E+06**	C5	70.5	66.2	C6	**2.8E+06**	
	**2.6E+06**	C5	70.5	65.9	C4	**2.8E+06**	
Ara 1	**7.4E+06**	C1	110.3	82.4	C2	**4.4E+06**	poly
	7.1E+06	C2	82.4	77.6	C3	**1.4E+07**	
	**8.1E+06**	C4	81.8	77.6	C3	**7.3E+06**	
	1.5E+07	C4	81.8	61.8	C5	**1.4E+07**	
Ara 2	**7.4E+06**	C1	110.1	82.2	C2	**4.4E+06**	poly
	7.1E+06	C2	82.2	77.7	C3	1.4E+07	
	**8.1E+06**	C4	81.7	77.7	C3	**7.3E+06**	
	1.5E+07	C4	81.7	61.8	C5	**1.4E+07**	
Ara 3	2.6E+07	C1	109.8	81.9	C2	**2.2E+07**	poly
	**8.1E+06**	C2	81.9	77.2	C3	**7.3E+06**	
	**8.1E+06**	C4	81.4	77.2	C3	**7.3E+06**	
	1.5E+07	C4	80.7	66.2	C5	**4.9E+06**	
Ara 4	2.6E+07	C1	109.8	81.8	C2	**2.2E+07**	poly
	**3.0E+06**	C2	81.8	75.0	C3	**9.9E+06**	
	**1.3E+06**	C4	81.7	74.9	C3	**9.9E+06**	
	1.6E+07	C4	81.3	67.2	C5	2.4E+07	
Gal 1	**1.0E+07**	C1	103.5	72.1	C2	**6.2E+06**	poly
	**2.8E+07**	C3	75.4	72.1	C2	**3.7E+07**	
	**1.8E+07**	C3	75.4	67.7	C4	**2.2E+07**	
	9.5E+06	C5	72.2	67.7	C4	**3.2E+06**	
	**7.4E+06**	C5	72.2	61.8	C6	**1.8E+07**	
Gal 2	3.3E+07	C1	98.5	72.4	C2	2.3E+07	mono
	**1.2E+07**	C3	73.7	72.4	C2	**4.0E+06**	
	**4.4E+07**	C3	73.7	71.7	C4	**4.8E+07**	
	6.3E+06	C5	75.1	71.7	C4	3.3E+06	
	**2.6E+06**	C5	75.1	62.9	C6	2.2E+07	
Gal 3	1.9E+06	C1	105.8	73.3	C2	2.4E+06	poly

	1.0E+05	C3	75.0	73.3	C2	4.1E+05	
	**1.3E+06**	C4	81.1	75.0	C3	**9.9E+06**	
	**1.3E+06**	C4	81.1	74.8	C5	**9.9E+06**	
	**7.2E+06**	C5	74.8	64.8	C6	**3.2E+06**	
Ac-Met 1	3.0E+06	Met	175.5	79.4	Ac	1.1E+06	
Ac-Met 2	1.4E+07	Met	175.2	77.6	Ac	7.7E+06	
Ac-Met 3	4.0E+06	Met	173.4	78.8	Ac	1.5E+06	
Ac-Met 4	9.8E+06	Met	175.6	76.5	Ac	4.5E+06	
Rha 1	**2.8E+06**	C1	100.4	77.1	C2	**2.7E+06**	poly
	**3.6E+06**	C2	77.1	70.2	C3	**3.6E+06**	
	**3.7E+06**	C4	71.7	70.2	C3	**5.8E+06**	
	**3.7E+06**	C4	71.7	70.3	C5	**5.8E+06**	
	2.8E+06	C5	70.3	17.8	C6	1.0E+07	
Rha 2	**2.8E+06**	C1	100.4	77.1	C2	**2.7E+06**	poly
	**3.6E+06**	C2	77.1	70.2	C3	**3.6E+06**	
	**3.7E+06**	C4	71.9	70.2	C3	**5.8E+06**	
	**3.7E+06**	C4	71.9	69.8	C5	**5.8E+06**	
	2.8E+06	C5	69.8	17.6	C6	1.0E+07	
Rha 3	**2.6E+06**	C1	100.4	69.6	C2	1.2E+06	poly
	**6.7E+06**	C3	78.7	70.2	C2	9.3E+06	
	**1.6E+06**	C3	78.7	73.6	C4	**1.8E+06**	
	**4.4E+07**	C4	73.6	70.9	C5	**4.8E+07**	
	3.0E+06	C5	70.9	17.4	C6	3.1E+06	
Glc 1	2.6E+06	C1	100.4	69.6	C2	2.6E+06	poly
	1.6E+06	C3	74.1	69.6	C2	4.5E+06	
	**1.2E+07**	C3	74.2	72.5	C4	**4.0E+06**	
	2.6E+06	C4	72.5	70.9	C5	1.3E+06	
	1.3E+07	C5	70.9	63.6	C6	1.5E+07	
Glc 2	2.9E+06	C1	100.0	76.8	C2	2.5E+06	poly
	**5.1E+06**	C2	76.8	71.3	C3	**4.9E+06**	
	1.3E+07	C3	71.3	63.8	C4	1.5E+07	
	1.3E+07	C5	71.8	63.8	C4	1.5E+07	
	**7.4E+06**	C5	71.8	61.9	C6	**8.8E+06**	
Xyl 1	1.0E+06	C1	97.7	76.1	C2	5.4E+06	mono

	**2.0E+06**	C3	77.2	76.1	C2	4.7E+06	
	**2.4E+07**	C3	77.2	67.7	C4	**2.2E+07**	
	**3.2E+07**	C4	67.7	60.2	C5	**2.2E+07**	
Xyl 2	1.0E+06	C1	100.3	70.8	C2	1.6E+06	poly
	**6.0E+06**	C3	75.9	74.8	C4	**7.0E+06**	
	1.4E+06	C3	75.9	70.8	C2	1.3E+06	
	**7.2E+06**	C4	74.8	64.9	C5	**3.2E+06**	
Xyl 3	1.3E+06	C1	100.9	77.4	C2	**1.3E+06**	poly
	**2.0E+06**	C2	77.4	75.1	C3	1.2E+06	
	1.5E+06	C4	79.5	75.1	C3	1.3E+06	
	1.3E+06	C4	79.5	67.8	C5	1.2E+06	
Unk 1	2.7E+06	C1	102.4	71.8	C2	4.6E+06	poly
	3.7E+06	C2	71.8	69.8	C3	5.8E+06	
	**4.4E+06**	C4	75.9	69.8	C3	4.5E+06	
	**5.8E+06**	C4	75.9	62.1	C5	**1.0E+07**	
Unk 2	**2.8E+06**	C1	100.2	76.9	C2	**2.7E+06**	poly
	**5.1E+06**	C2	76.9	71.4	C3	**4.9E+06**	
	2.0E+07	C4	83.7	71.4	C3	5.5E+07	
	3.2E+07	C4	83.7	61.7	C5	3.8E+07	

Supplementary Table 6. Results of the ssNMR-based glycan quantification. SsNMR can quantify the total amount of each glycan but also its relative abundance as mono- or polysaccharides, according to their C_1 chemical shifts. Glycan quantification has been performed as described before[16]. Briefly, quantification takes into account all the carbon peak integrals corresponding to one glycan type (values in black and orange in **Supplementary Table 5**). Then integrals are normalized using the number of carbon integrated (*tot nC*) and, for comparison with other glycans, we take into account the number of spin system assigned (*n spin syst.*). Results of the GC/MS-based quantification are shown for comparison in the last column.

		sum int.	nC	nC overlap	tot nC	int/nC_{total}	n spin syst.	$(int/nC)*n_{spin\ system}$	% norm.	GC/MS
Man	TOTAL	8.9E+08	63	47	110	8.1E+06	14	1.1E+08	48.2	45
	poly.	5.8E+08	47	38	85	6.8E+06	10	6.8E+07	29.0	
	mono.	3.1E+08	16	9	25	1.2E+07	4	5.0E+07	21.3	
Ara	TOTAL	1.6E+08	10	9	19	8.2E+06	4	3.3E+07	14.0	16
	poly.	1.6E+08	10	9	19	8.2E+06	4	3.3E+07	14.0	
	mono.	0.0E+00	0	0	0	0.0E+00	0	0.0E+00	0.0	
GlcNAc	TOTAL	4.6E+07	8	0	8	5.7E+06	4	2.3E+07	9.7	5.6
	poly.	4.6E+07	8	0	8	5.7E+06	4	2.3E+07	9.7	
	mono.	0.0E+00	0	0	0	0.0E+00	0	0.0E+00	0.0	
Gal	TOTAL	1.6E+08	10	0	10	1.6E+07	3	4.8E+07	20.4	17
	poly.	1.4E+07	5	0	5	2.9E+06	2	5.7E+06	2.4	
	mono.	1.5E+08	5	0	5	2.9E+07	1	2.9E+07	12.4	
Rha	TOTAL	2.0E+07	5	2	7	2.9E+06	2	5.8E+06	2.5	2
	poly.	2.0E+07	5	2	7	2.9E+06	2	5.8E+06	2.5	
	mono.	0.0E+00	0	0	0	0.0E+00	0	0.0E+00	0.0	
Glc	TOTAL	4.9E+07	10	4	14	3.5E+06	2	6.9E+06	3.0	1.3
	poly.	4.9E+07	10	4	14	3.5E+06	2	6.9E+06	3.0	
	mono.	0.0E+00	0	0	0	0.0E+00	0	0.0E+00	0.0	
Xyl	TOTAL	2.6E+07	14	1	15	1.7E+06	3	5.2E+06	2.2	1.3
	poly.	1.3E+07	10	1	11	1.2E+06	2	2.4E+06	1.0	
	mono.	1.3E+07	4	0	4	3.3E+06	1	3.3E+06	1.4	

Supplementary Table 7. ^{13}C glycan composition of different intact *C. reinhardtii* strains. Highlighted column (*bald2* cell-wall extract) corresponds to the cell-wall extract reported in the main text. The strains reported are the wild-type (*wt*) strain, strain without flagella (*bald2*), cell-wall deprived (*cw15*) and starchless mutants. The reported ^{13}C alditol values show relative mole percentages (%).

Strain / ^{13}C alditol	*bald2* whole cell	*bald2* cw extracted	*bald2* cw extract	*wt* whole cell	*wt* cw extracted	*wt* cw extract	*cw15* whole cell	*cw15* cw extracted	sta6-1 whole cell	sta6-1 cw extracted
Man	27.2	12.8	50.4	12.9	4.9	48.4	2.4	10.5	28.6	12.8
Ara	22.9	24.8	20.1	13.3	11.8	19.6	9.5	10.2	24.9	30.6
Gal	15.9	14.2	18.7	11.6	9.8	20.0	1.7	7.7	29.8	34.0
Rha	4.9	6.5	2.2	0.9	0.6	2.6	0.0	0.7	3.2	5.1
Glc	22.7	35.9	1.5	54.4	66.2	2.0	85.1	66.0	3.8	5.6
Xyl	1.7	1.9	1.5	1.6	1.7	1.5	1.0	1.6	2.2	4.7
GlcNAc	4.7	4.0	5.6	5.2	5.0	5.9	0.2	3.3	7.5	7.1

Supplementary Table 8. Glycan content determined by GC-MS.

	Whole cell	Whole cell after wall extraction	Cell wall extract	Proportionality factor
Man	27.2	12.8	50.4	38
Ara	22.9	24.8	20.1	40
Gal	15.9	14.2	18.7	38
Rha	4.9	6.5	2.2	37
Glc	22.7	35.9	1.5	38
Xyl	1.7	1.9	1.5	50
GlcNAc	4.7	4.0	5.6	44

Glycan contents (in molar %) of cells before and after wall extraction, and in the extracted cell wall. The proportionality factor corresponds to the cell wall contribution (molar %) in the whole cell glycans. See **Supplementary Table 7** for glycan quantification in other samples.

We calculated the contribution of glycans in the whole cells that originate from the cell wall, and found a similar proportionality factor for each glycan, confirming that almost no sugar units were lost or altered during the extraction and reconstitution, with an uncertainty for xylose, which proportion is close to ssNMR and MS detection limits. This proportionality factor indicates that about 40% of the whole cell glycans are contained in the cell wall.

Supplementary Table 9. ^{13}C ssNMR amino acid assignment of *C. reinhardtii bald2* cell wall. Amino acid assignment is performed using 800 MHz DP-based *J*-INADEQUATE and DARR spectra. ^{13}C chemical shifts are referenced using TMS (by setting adamantane CH_2 chemical shift to 38.48 ppm), therefore TALOS+ calculation have been made shifting all the chemical shifts by 2.0 ppm to match DSS referencing. "b" and "c" correspond to beta-sheet and random-coil/polyproline helix prediction given by TALOS+ calculation, respectively.

Amino acids residues	secondary structure	Co	Cα	Cβ	Cγ	Cδ	Cε	Cζ
Lys1	b	174.0	53.9	30.7	22.5	26.7	41.9	
Lys2	b	174.3	54.0	31.9	23.1	27.7	40.6	
Lys3	b	174.8	54.3	31.2	22.9	27.2	40.4	
Lys4	b	174.3	54.1	33.3	22.3	26.9	40.1	
Asp1	b	174.0	54.0	40.9	178.9			
Asp2	c	174.0	54.0	40.3	177.8			
Asp3	c	175.6	53.6	39.3	178.2			
Asp4	c	175.2	54.1	37.6	178.3			
Asn1	c	174.2	52.6	39.3	176.0			
Asn2	c	174.3	52.3	38.6	176.4			
Asn3	c	174.4	51.1	37.2	175.2			
Glu1	b	173.9	55.8	29.4	33.8	182.9		
Glu2	c	179.1	54.0	25.4	30.1	182.7		
Glu3	b	174.4	54.1	29.2	34.9	182.6		
Glu4	c	175.2	55.1	28.3	34.4	181.8		
Gln1	c	175.2	54.6	28.4	32.5	179.0		
Gln2	b	174.3	53.7	30.2	31.0	178.6		
Gln3	b	174.1	53.9	27.6	32.0	178.4		
Gln4	c	175.5	55.7	29.9	33.3	178.1		
Gln5	b	174.5	54.4	30.5	32.0	178.4		
Hyp1	u	171.9	58.8	34.8	73.1	50.2		
Pro1	c	175.3	60.6	30.5	25.7	49.0		
Pro2	c	175.4	61.3	30.1	25.4	48.5		
Pro3	c	174.0	59.2	29.0	25.2	47.9		
Pro4	c	175.8	61.7	28.5	25.0	47.4		
Pro5	c	175.9	62.0	29.8	24.6	47.1		
Arg1	b	174.4	54.2	31.5	24.9	40.6		
Arg2	b	175.6	53.5	30.4	25.8	37.7		
Arg3	b	173.9	53.9	30.7	24.9	40.6		
Arg4	b	175.6	53.7	30.1	25.2	41.6		
Arg5	c	173.9	55.8	29.8	24.6	41.6		
Arg6	b	174.4	54.2	28.9	25.4	41.6		
Arg7	c	176.5	54.6	28.5	25.0	40.4		
Se1	b	172.7	56.6	64.4				
Ser2	c	175.6	57.6	64.1				
Ser3	b	172.7	56.6	63.9				
Ser4	c	176.9	58.1	63.2				
Ser5	c	176.1	56.6	62.1				
Thr1	b	174.1	60.4	68.0	19.6			
Thr2	b	173.9	61.1	68.9	19.4			
Thr3	b	173.9	59.9	70.8	17.5			

Gly1	b	169.9	42.6					
Gly2	c	171.1	43.4					
Gly3	c	172.0	43.3					
Gly4	c	172.8	43.6					
Gly5	c	173.2	42.6					
Ala1	b	173.7	49.0	25.7				
Ala2	b	173.6	48.5	25.4				
Ala3	b	/	47.9	25.2				
Ala4	b	/	47.4	25.0				
Ala5	b	/	47.1	24.6				
Ala6	b	173.6	48.3	22.8				
Ala7	c	176.6	51.8	18.4				
Ala8	b	177.0	50.6	19.2				
Ala9	b	175.1	50.0	18.9				
Ala10	c	176.7	51.5	17.2				
Ala11	c	176.1	51.0	17.2				
Ala12	c	177.2	50.4	17.5				
Ala13	b	175.2	50.1	17.3				
Ala14	c	173.6	48.4	16.6				
Tyr1	c	174.6	51.1	37.4	130.2	131.6	116.7	156.4
Tyr2	c	174.6	51.1	37.4	130.2	131.2	116.9	155.5
Tyr3	c	174.6	51.1	37.4	130.2	131.6	116.0	156.0
Tyr4	c	174.6	51.1	37.4	130.2	131.6	116.5	155.1
Tyr5	c	174.6	51.1	37.4	130.2	131.6	115.9	155.5
Leu1	c	175.8	54.9	41.8	26.6	21.5		
Leu2	c	175.2	55.0	41.5	25.4	21.9		
Leu3	b	174.0	54.2	40.8	25.1	23.2		
Leu4	c	176.2	53.8	39.9	27.0	22.2		
Leu5	c	176.6	53.3	40.5	27.2	22.9		
Leu6	b	175.9	53.3	40.6	27.8	23.3		
Leu7	c	176.6	52.4	39.7	28.8	23.0		
Met1	c	175.6	54.7	30.6	32.0	19.0		
Met2	c	176.4	53.6	30.2	31.0	18.5		
Met3	c	176.2	53.8	30.7	29.7	19.0		
Met4	c	174.7	54.4	30.9	29.8	17.6		
Val1	c	175.8	62.1	29.8	17.7			
Val2	c	175.6	62.3	29.7	19.0			
Val3	b	174.8	61.5	31.4	19.7			
Val4	b	174.8	61.2	30.3	19.5			
Val5	b	172.1	60.7	30.1	18.4			
Val6	b	172.4	60.1	30.9	18.5			
Ile1	b	173.9	59.3	36.7	25.2	11.1		
					15.8			
Ile2	b	174.2	59.3	36.7	25.4	11.8		
					15.8			
Ile3	b	173.7	59.2	36.9	25.5	12.0		
					15.7			
Ile4	b	/	/	37.7	/	/		
					16.3			
Phe1	u	/	/	138.6	130.2	129.3	127.6	
Phe2	u	/	/	138.6	130.1	129.4	128.1	

Supplementary Table 10. Integrals of the amino acid used for their quantification. Values in red correspond to amino-acid peaks overlapping with other types of amino acids, and therefore cannot be used for quantification, while overlapping peaks among same kinds of residues, in orange, can be used for quantification. This table also allows to see the TALOS+ secondary structure prediction.

	peak int.		SQ	SQ		peak int.	TALOS second. Struct.
Ala 1	1.3E+07	Ca	49.0	25.7	Cb	1.1E+07	Beta-sheet
	**5.7E+06**	Co	173.7	49.0	Ca	**5.5E+06**	
Ala 2	1.3E+07	Ca	48.5	25.4	Cb	1.6E+07	Beta-sheet
	**5.7E+06**	Co	173.6	48.5	Ca	**5.5E+06**	
Ala 3	5.5E+06	*Ca*	47.9	25.2	*Cb*	9.5E+06	Beta-sheet
Ala 4	1.4E+07	*Ca*	47.4	25.0	*Cb*	4.7E+06	Beta-sheet
Ala 5	1.6E+07	*Ca*	47.1	24.6	*Cb*	1.2E+07	Beta-sheet
Ala 6	5.9E+06	Ca	48.3	22.8	Cb	5.2E+06	Beta-sheet
	**5.7E+06**	Ca	173.6	48.3	Ca	**5.5E+06**	
Ala 7	1.4E+07	Ca	51.8	18.4	Cb	3.9E+07	Random coil
	1.1E+07	Co	176.6	51.8	Ca	1.9E+06	
Ala 8	9.8E+05	Ca	50.6	19.2	Cb	1.1E+07	Beta-sheet
	**7.6E+06**	Co	177.0	50.6	Ca	**4.2E+06**	
Ala 9	7.6E+06	Ca	50.0	18.9	Cb	2.3E+07	Beta-sheet
	**3.6E+07**	Co	175.1	50.0	Ca	**3.0E+06**	
Ala 10	1.0E+07	Ca	51.5	17.2	Cb	1.6E+07	Random coil
	**2.7E+07**	Co	176.7	51.5	Ca	**1.9E+07**	
Ala 11	1.6E+06	Ca	51.0	17.2	Cb	3.2E+07	Random coil
	**2.7E+07**	Co	176.1	51.0	Ca	**1.9E+07**	
Ala 12	**5.8E+06**	Ca	50.4	17.5	Cb	**1.1E+07**	Random coil
	**7.6E+06**	Co	177.2	50.4	Ca	**4.2E+06**	
Ala 13	**5.8E+06**	Ca	50.1	17.3	Cb	**1.1E+07**	Beta-sheet
	**3.6E+07**	Co	175.2	50.1	Ca	**3.0E+06**	
Ala 14	5.5E+06	Ca	48.4	16.6	Cb	1.2E+07	Random coil
	**5.7E+06**	Co	173.6	48.4	Ca	**5.5E+06**	
Arg 1	**2.6E+07**	Ca	54.2	31.5	Cb	**4.4E+07**	Beta-sheet
	3.1E+06	Cb	31.5	24.9	Cc	3.2E+06	
	**2.7E+07**	Cd	40.6	24.9	Cc	**2.4E+07**	
	**9.0E+07**	Co	174.4	54.2	Ca	**9.9E+07**	
Arg 2	**3.1E+06**	Ca	53.5	30.4	Cb	**3.6E+06**	Beta-sheet
	**2.4E+06**	Cb	30.4	25.8	Cc	**1.3E+07**	
	5.6E+06	Cd	37.7	25.8	Cc	5.6E+06	
	**6.2E+06**	Co	175.6	53.5	Ca	**1.2E+07**	
Arg 3	**2.6E+07**	Ca	53.9	30.7	Cb	**4.4E+06**	Beta-sheet
	1.8E+06	Cb	30.7	24.9	Cc	1.6E+06	
	**2.7E+07**	Cd	40.6	24.9	Cc	**2.4E+07**	
	**9.0E+07**	Co	173.9	53.9	Ca	**9.9E+07**	
Arg 4	**3.1E+06**	Ca	53.7	30.1	Cb	**3.6E+06**	Beta-sheet
	**2.8E+07**	Cb	30.1	25.2	Cc	**3.1E+07**	
	**8.5E+07**	Cd	41.6	25.2	Cc	**6.9E+07**	
	**6.2E+06**	Co	175.6	53.7	Ca	**1.2E+07**	

Arg 5	**7.4E+06**	Ca	55.8	29.8	Cb	**1.6E+07**	Random coil
	7.0E+06	Cb	29.8	24.6	C	5.3E+06	
	**8.5E+07**	Cd	41.6	24.6	Cc	**6.9E+07**	
	**2.4E+06**	Co	173.9	55.8	Ca	**3.2E+06**	
Arg 6	**4.5E+06**	Ca	54.2	28.9	Cb	**7.5E+06**	Beta-sheet
	1.3E+07	Cb	28.9	25.4	Cc	1.5E+07	
	**8.5E+07**	Cd	41.6	25.4	Cc	**6.9E+07**	
	**9.0E+07**	Co	174.4	54.2	Ca	**9.9E+07**	
Arg 7	**3.3E+07**	Ca	54.6	28.5	Cb	**5.5E+07**	Random coil
	**3.2E+06**	Cb	28.5	25.0	Cc	**7.6E+06**	
	**2.7E+07**	Cd	40.4	25.0	Cc	**2.4E+07**	
	1.5E+07	Co	176.5	54.6	Ca	**3.6E+07**	
Asn 1	**2.6E+07**	Ca	52.6	39.3	Cb	**1.9E+07**	Random coil
	9.4E+06	Cc	176.0	39.3	Cb	4.0E+06	
	2.6E+07	Co	174.2	52.6	Ca	4.3E+07	
Asn 2	2.2E+06	Ca	52.3	38.6	Cb	4.6E+06	Random coil
	5.7E+06	Cc	176.4	38.6	Cb	4.6E+06	
	2.6E+07	Co	174.3	52.3	Ca	4.3E+07	
Asn 3	**9.1E+06**	Ca	51.1	37.2	Cb	**2.9E+07**	Random coil
	4.6E+07	Cc	175.2	37.2	Cb	2.9E+07	
	**3.6E+07**	Co	174.4	51.1	Ca	**1.3E+07**	
Asp 1	6.7E+06	Ca	54.0	40.9	Cb	9.5E+06	Beta-sheet
	1.4E+07	Cc	178.9	40.9	Cb	**1.3E+07**	
	**9.0E+07**	Co	174.0	54.0	Ca	**9.9E+07**	
Asp 2	7.9E+06	Ca	53.1	40.3	Cb	**1.2E+07**	Random coil
	6.7E+06	Cc	177.8	40.3	Cb	**1.2E+07**	
	**9.0E+07**	Co	174.0	54.0	Ca	**9.9E+07**	
Asp 3	2.0E+06	Ca	53.6	39.3	Cb	**3.1E+07**	Random coil
	5.8E+07	Cc	178.2	39.3	Cb	**3.1E+07**	
	**6.2E+06**	Co	175.6	53.6	Ca	**1.2E+07**	
Asp 4	2.0E+06	Ca	54.1	37.6	Cb	**3.1E+07**	Random coil
	1.5E+07	Cc	178.3	37.6	Cb	**3.5E+06**	
	**9.0E+07**	Co	175.2	54.1	Ca	**9.9E+07**	
Gln 1	**3.3E+07**	Ca	54.6	28.4	Cb	**5.5E+07**	Random coil
	9.1E+06	Cc	32.5	28.4	Cb	1.2E+07	
	1.1E+07	Cd	179.0	32.5	Cc	**8.2E+06**	
	**9.0E+07**	Co	175.2	54.2	Ca	**9.9E+07**	
Gln 2	**3.1E+06**	Ca	53.7	30.2	Cb	**3.6E+06**	Beta-sheet
	**7.3E+06**	Cc	31.0	30.2	Cb	**1.2E+07**	
	**5.6E+07**	Cd	178.6	31.0	Cc	4.0E+07	
	**9.0E+07**	Co	174.3	54.2	Ca	**9.9E+07**	
Gln 3	1.4E+07	Ca	53.9	27.6	Cb	1.9E+07	Beta-sheet
	2.7E+07	Cc	32.0	27.6	Cb	2.7E+07	
	**5.6E+07**	Cd	178.4	32.0	Cc	4.0E+07	
	**9.0E+07**	Co	174.1	54.0	Ca	**9.9E+07**	
Gln 4	**7.4E+06**	Ca	55.7	29.9	Cb	**1.6E+07**	Random coil
	1.4E+06	Cc	33.3	29.9	Cb	1.8E+06	
	1.1E+07	Cd	178.1	33.3	Cc	5.9E+06	
	2.7E+07	Co	175.5	55.7	Ca	1.0E+07	
Gln 5	**2.6E+07**	Ca	54.4	30.5	Cb	**4.4E+06**	Beta-sheet

	**6.5E+06**	Cc	32.0	30.5	Cb	**1.0E+07**	
	**5.6E+07**	Cd	178.4	32.0	Cc	4.0E+07	
	**9.0E+07**	Co	174.5	54.4	Ca	**9.9E+07**	
Glu 1	7.4E+06	Ca	55.8	29.4	Cb	1.6E+07	Beta-sheet
	5.7E+06	Cc	33.8	29.4	Cb	**1.7E+07**	
	2.3E+07	Cd	182.9	33.8	Cc	4.2E+06	
	**2.4E+06**	Co	173.9	55.8	Ca	**3.2E+06**	
Glu 2	2.0E+06	Ca	54.0	25.4	Cb	2.2E+06	Random coil
	**2.8E+07**	Cc	30.1	25.4	Cb	**3.1E+07**	
	9.0E+06	Cd	182.7	30.1	Cc	8.0E+06	
	2.6E+06	Co	179.1	54.0	Ca	7.3E+06	
Glu 3	**4.5E+06**	CA	54.1	29.1	Cb	**7.5E+06**	Beta-sheet
	6.0E+06	Cc	34.9	29.2	Cb	**1.7E+07**	
	2.4E+07	Cd	182.6	34.9	Cc	1.5E+07	
	**9.0E+07**	Co	174.4	54.1	Ca	**9.9E+07**	
Glu 4	7.6E+06	Ca	55.1	28.3	Cb	8.7E+06	Random coil
	3.3E+07	Cc	34.4	28.3	Cb	4.6E+07	
	1.5E+08	Cd	181.8	34.4	Cc	7.6E+07	
	**3.5E+07**	Co	175.2	55.1	Ca	**1.4E+07**	
Gly 1	5.3E+06	Co	169.9	42.6	Ca	6.9E+06	Beta-sheet
Gly 2	1.9E+07	Co	171.1	43.4	Ca	9.7E+06	Random coil
Gly 3	3.5E+07	Co	172.0	43.3	Ca	1.9E+07	Random coil
Gly 4	3.5E+07	Co	172.8	43.6	Ca	2.9E+07	Random coil
Gly 5	2.8E+06	Co	173.2	42.6	Ca	1.2E+06	Random coil
Ile 1	**6.9E+06**	Ca	59.3	36.7	Cb	**4.3E+06**	Beta-sheet
	**1.3E+07**	Cb	36.7	25.2	Cc1	**1.7E+07**	
	**6.6E+06**	Cb	36.7	15.8	Cc2	**1.5E+07**	
	**1.4E+07**	Cc1	25.2	11.1	Cd	2.7E+07	
	**1.4E+06**	Co	173.9	59.3	Ca	**1.3E+07**	
Ile 2	**6.9E+06**	Ca	59.3	36.7	Cb	**4.3E+06**	Beta-sheet
	**1.3E+07**	Cb	36.7	25.4	Cc1	**1.7E+07**	
	**6.6E+06**	Cb	36.7	15.8	Cc2	**1.5E+07**	
	**1.4E+07**	Cc1	25.4	11.8	Cd	**8.5E+06**	
	**1.4E+06**	Co	174.2	59.3	Ca	**1.3E+07**	
Ile 3	**6.9E+06**	Ca	59.2	36.9	Cb	**4.3E+06**	Beta-sheet
	**1.3E+07**	Cb	36.9	25.5	Cc1	**1.7E+07**	
	**6.6E+06**	Cb	36.9	15.7	Cc2	**1.5E+07**	
	**1.4E+07**	Cc1	25.5	12.0	Cd	**8.5E+06**	
	**1.4E+06**	Co	173.7	59.2	Ca	**1.3E+07**	
Ile 4	4.8E+06	Cb	37.7	16.3	Cb	1.6E+07	Beta-sheet
Leu 1	6.0E+06	Ca	54.9	41.8	Cb	8.9E+06	Random coil
	5.3E+06	Cb	41.8	26.6	Cc	1.6E+06	
	1.6E+06	Cc	26.6	21.5	Cd	1.1E+07	
	3.0E+07	Co	175.8	54.9	Ca	**3.6E+07**	
Leu 2	**4.8E+06**	Ca	55.0	41.5	Cb	**1.3E+07**	Random coil
	**8.5E+07**	Cb	41.5	25.4	Cc	**6.9E+07**	
	5.9E+06	Cc	25.4	21.9	Cd	1.2E+07	
	**3.5E+07**	Co	175.2	55.0	Ca	**1.4E+07**	
Leu 3	6.7E+06	Ca	54.2	40.8	Cb	3.5E+06	Beta-sheet
	**2.7E+07**	Cb	40.8	25.1	Cc	**2.4E+07**	

	7.0E+07	Cc	25.1	23.2	Cd	1.6E+07	
	**9.0E+07**	Co	174.0	54.2	Ca	**9.9E+07**	
Leu 4	7.9E+06	Ca	53.8	39.9	Cb	**1.2E+07**	Random coil
	**1.7E+08**	Cb	39.9	27.0	Cc	**1.1E+08**	
	**5.9E+06**	Cc	27.0	22.2	Cd	**2.1E+07**	
	**6.2E+06**	Co	176.2	53.8	Ca	**1.2E+07**	
Leu 5	2.7E+06	Ca	53.3	40.5	Cb	3.1E+07	Random coil
	**1.7E+08**	Cb	40.5	27.2	Cc	**1.1E+08**	
	**7.0E+07**	Cc	27.2	22.9	Cd	**6.0E+07**	
	**6.2E+06**	Co	176.6	53.3	Ca	**1.2E+07**	
Leu 6	2.7E+06	Ca	53.3	40.6	Cb	3.1E+07	Beta-sheet
	1.9E+07	Cb	40.6	27.8	Cc	1.0E+07	
	**3.5E+06**	Cc	27.8	23.3	Cd	**1.5E+06**	
	1.8E+07	Co	175.9	53.3	Ca	3.0E+06	
Leu 7	**2.6E+07**	Ca	52.4	39.7	Cb	**1.9E+07**	Random coil
	1.2E+07	Cb	39.7	28.8	Cc	2.0E+06	
	4.7E+06	Cc	28.8	23.0	Cd	9.9E+05	
	1.3E+07	Co	176.6	52.4	Ca	1.1E+07	
Lys 1	**2.6E+07**	Ca	53.9	30.7	Cb	**4.4E+06**	Beta-sheet
	5.4E+06	Cb	30.7	22.5	Cc	5.7E+06	
	**5.9E+06**	Cd	26.7	22.5	Cc	**2.1E+07**	
	5.5E+06	Ce	41.9	26.7	Cd	2.9E+06	
	**9.0E+07**	Co	174.0	53.9	Ca	**9.9E+07**	
Lys 2	**2.6E+07**	Ca	54.0	31.9	Cb	4.0E+07	Beta-sheet
	9.4E+06	Cb	31.9	23.1	Cc	3.8E+06	
	**3.5E+06**	Cd	27.7	23.1	Cc	**1.5E+06**	
	1.9E+07	Ce	40.6	27.7	Cd	1.0E+07	
	**9.0E+07**	Co	174.3	54.0	Ca	**9.9E+07**	
Lys 3	**2.6E+07**	Ca	54.3	31.2	Cb	**4.4E+07**	Beta-sheet
	4.2E+07	Cb	31.2	22.9	Cc	5.1E+07	
	**7.0E+07**	Cd	27.2	22.9	Cc	**6.0E+07**	
	**1.7E+08**	Ce	40.4	27.2	Cd	**1.1E+08**	
	**9.0E+07**	Co	174.8	54.3	Ca	**9.9E+07**	
Lys 4	4.7E+04	Ca	54.1	33.3	Cb	**5.9E+06**	Beta-sheet
	6.5E+06	Cb	33.3	22.3	Cc	4.6E+06	
	**5.9E+06**	Cd	26.9	22.3	Cc	**2.1E+07**	
	**1.7E+08**	Ce	40.1	26.9	Cd	**1.1E+08**	
	**9.0E+07**	Co	174.3	54.1	Ca	**9.9E+07**	
Met 1	**2.6E+07**	Ca	54.7	30.6	Cb	**4.4E+06**	Random coil
	**6.5E+06**	Cc	32.0	30.6	Cb	**1.0E+07**	
	3.4E+06	Cc	32.0	19.0	Cd	1.3E+07	
	1.9E+06	Co	175.6	54.7	Ca	**3.6E+07**	
Met 2	**3.1E+06**	Ca	53.6	30.2	Cb	**3.6E+06**	Random coil
	**7.3E+06**	Cc	31.0	30.2	Cb	**1.2E+07**	
	**6.1E+07**	Cc	31.0	18.5	Cd	**6.3E+07**	
	**6.2E+06**	Co	176.4	53.6	Ca	**1.2E+07**	
Met 3	**2.6E+07**	Ca	53.8	30.7	Cb	**4.4E+06**	Random coil
	**7.3E+06**	Cb	30.7	29.7	Cc	**1.2E+07**	
	**2.6E+06**	Cc	29.7	19.0	Cd	**1.3E+07**	
	**6.2E+06**	Co	176.2	53.8	Ca	**1.2E+07**	

Met 4	**2.6E+07**	Ca	54.4	30.9	Cb	**4.4E+07**	Random coil
	**7.3E+06**	Cb	30.9	29.8	Cc	**1.2E+07**	
	**4.4E+06**	Cc	29.8	17.6	Cd	**9.7E+06**	
	**9.0E+07**	Co	174.7	54.4	Ca	**9.9E+07**	
Phe 1-u	2.0E+06	Cc	138.6	130.2	Cd	6.3E+06	Unknown
	3.1E+06	Cd	130.2	129.3	Ce	1.7E+06	
	7.1E+06	Ce	129.3	127.6	Cf	4.2E+06	
Phe 2-u	2.0E+06	Cc	138.6	130.1	Cd	6.3E+06	Unknown
	1.7E+06	Cd	130.1	129.4	Ce	1.7E+06	
	5.9E+06	Ce	129.4	128.1	Cf	5.0E+06	
Pro 1	1.8E+07	Ca	60.6	30.5	Cb	1.9E+07	Random coil
	**2.4E+06**	Cb	30.5	25.7	Cc	**1.3E+07**	
	1.3E+07	Cd	49.0	25.7	Cc	1.1E+07	
	4.5E+06	Co	175.3	60.6	Ca	2.3E+07	
Pro 2	1.8E+07	Ca	61.3	30.1	Cb	2.2E+07	Random coil
	2.8E+07	Cb	30.1	25.4	Cc	3.1E+07	
	2.6E+07	Cd	48.5	25.4	Cc	3.1E+07	
	3.1E+06	Co	175.4	61.3	Ca	2.1E+07	
Pro 3	2.2E+06	Ca	59.2	29.0	Cb	2.1E+06	Random coil
	2.6E+07	Cb	29.0	25.3	Cc	3.0E+07	
	5.5E+06	Cd	47.9	25.2	Cc	9.5E+06	
	**1.4E+06**	Co	174.0	59.2	Ca	**1.3E+07**	
Pro 4	1.5E+06	Ca	61.7	28.5	Cb	2.8E+06	Random coil
	3.2E+06	Cb	28.5	25.0	Cc	7.6E+06	
	1.4E+07	Cd	47.4	25.0	Cc	4.7E+06	
	3.1E+06	Co	175.8	61.7	Ca	2.1E+07	
Pro 5	5.3E+06	Ca	62.0	29.8	Cb	8.4E+06	Random coil
	7.0E+06	Cb	29.8	24.6	Cc	5.3E+06	
	1.6E+07	Cd	47.1	24.6	Cc	1.2E+07	
	2.7E+07	Co	175.9	62.0	Ca	7.8E+06	
Ser 1	1.1E+07	Cb	64.4	56.5	Ca	1.0E+07	Beta-sheet
	3.3E+07	Co	172.7	56.6	Ca	1.9E+07	
Ser 2	1.5E+06	Cb	64.1	57.6	Ca	8.3E+05	Random coil
	1.1E+07	Co	175.6	57.6	Ca	7.2E+06	
Ser 3	6.5E+06	Cb	63.9	56.2	Ca	3.1E+06	Beta-sheet
	3.3E+07	Co	172.7	56.6	Ca	1.9E+07	
Ser 4	8.5E+06	Cb	63.2	58.1	Ca	4.4E+06	Random coil
	7.6E+06	Co	176.9	58.1	Ca	3.5E+06	
Ser 5	6.3E+07	Cb	62.1	56.6	Ca	3.1E+07	Random coil
	3.5E+06	Co	176.1	56.6	Ca	1.1E+07	
Thr 1	**3.2E+07**	Cb	68.0	60.4	Ca	**2.2E+07**	Beta-sheet
	4.4E+07	Cb	68.0	19.6	Cc	1.9E+07	
	1.6E+07	Co	174.1	60.4	Ca	2.4E+07	
Thr 2	7.6E+06	Cb	68.9	61.1	Ca	3.1E+06	Beta-sheet
	1.4E+07	Cb	68.9	19.4	Cc	2.9E+07	
	2.7E+07	Co	173.9	61.1	Ca	2.8E+06	
Thr 3	1.9E+06	Cb	70.8	59.9	Ca	7.9E+06	Beta-sheet
	5.8E+06	Cb	70.8	17.5	Cc	1.3E+07	
	1.6E+07	Co	173.9	59.9	Ca	2.4E+07	
Tyr 1	**9.1E+06**	C1	51.1	37.4	Cb	**2.9E+07**	Random coil

	**4.0E+06**	Cc	130.2	37.4	Cb	**6.5E+06**	
	**3.4E+06**	Cd	131.6	130.2	Cc	**4.7E+06**	
	**1.4E+07**	Cd	131.6	116.7	Ce	**7.9E+06**	
	8.4E+06	Cf	156.4	116.7	Ce	2.9E+06	
	**3.6E+07**	Co	174.6	51.1	Ca	**1.3E+07**	
Tyr 2	**9.1E+06**	Ca	51.1	37.4	Cb	**2.9E+07**	Random coil
	**4.0E+06**	Cc	130.2	37.4	Cb	**6.5E+06**	
	4.4E+06	Cd	131.2	130.2	Cc	**4.7E+06**	
	**1.4E+07**	Cd	131.2	116.9	Ce	**7.9E+06**	
	3.8E+06	Cf	155.5	116.9	Ce	6.8E+06	
	3.6E+07	Co	174.6	51.1	Ca	**1.3E+07**	
Tyr 3	9.1E+06	Ca	51.1	37.4	Cb	**2.9E+07**	Random coil
	**4.0E+06**	Cc	130.2	37.4	Cb	**6.5E+06**	
	**3.4E+06**	Cd	131.6	130.2	Cc	**4.7E+06**	
	**1.4E+07**	Cd	131.6	116.0	Ce	**7.9E+06**	
	8.1E+06	Cf	156.0	116.0	Ce	6.0E+06	
	**3.6E+07**	Co	174.6	51.1	Ca	**1.3E+07**	
Tyr 4	**9.1E+06**	Ca	51.1	37.4	Cb	**2.9E+07**	Random coil
	**4.0E+06**	Cc	130.2	37.4	Cb	**6.5E+06**	
	**3.4E+06**	Cd	131.6	130.2	Cc	**4.7E+06**	
	**1.4E+07**	Cd	131.6	116.5	Ce	**7.9E+06**	
	8.3E+06	Cf	155.1	116.5	Ce	8.6E+06	
	**3.6E+07**	Co	174.6	51.1	Ca	**1.3E+07**	
Tyr 5	**9.1E+06**	Ca	51.1	37.4	Cb	**2.9E+07**	Random coil
	**4.0E+06**	Cc	130.2	37.4	Cb	**6.5E+06**	
	**3.4E+06**	Cd	131.6	130.2	Cc	**4.7E+06**	
	**1.4E+07**	Cd	131.6	115.9	Ce	**7.9E+06**	
	8.7E+06	Cf	155.5	115.9	Ce	8.5E+06	
	**3.6E+07**	Co	174.6	51.1	Ca	**1.3E+07**	
Val 1	2.0E+06	Ca	61.6	31.4	Cb	9.5E+06	Random coil
	1.1E+06	Cb	31.4	19.7	Cc	3.9E+06	
	**2.7E+07**	Co	175.8	62.1	Ca	**7.8E+06**	
Val 2	**5.3E+06**	Ca	62.1	29.8	Cb	**8.4E+06**	Random coil
	**4.4E+06**	Cb	29.8	17.7	Cc	**9.7E+06**	
	**2.7E+07**	Co	175.6	62.3	Ca	**7.8E+06**	
Val 3	**5.3E+06**	Ca	62.3	29.7	Cb	**8.4E+06**	Beta-sheet
	**2.6E+06**	Cb	29.7	19.0	Cc	**1.3E+07**	
	**5.0E+07**	Co	174.8	61.5	Ca	**1.0E+06**	
Val 4	1.5E+07	Ca	61.2	30.3	Cb	3.9E+06	Beta-sheet
	6.1E+06	Cb	30.3	19.5	Cc	6.8E+06	
	**5.0E+07**	Co	174.8	61.2	Ca	**1.0E+06**	
Val 5	**1.8E+07**	Ca	60.7	30.1	Cb	**2.2E+07**	Beta-sheet
	**2.6E+06**	Cb	30.1	18.4	Cc	1.8E+06	
	3.9E+07	Co	172.1	60.7	Ca	**3.1E+06**	
Val 7	**1.8E+07**	Ca	60.1	30.9	Cb	**1.9E+07**	Beta-sheet
	**6.1E+07**	Cb	30.9	18.5	Cc	**6.3E+07**	
	9.6E+06	Co	172.4	60.1	Ca	2.1E+06	

Supplementary Table 11. Results of the amino acid quantification. SSNMR can quantify the total amount of each amino acid, but also its relative abundance according to their predicted secondary structure. Note that TALOS+ predicts β-sheet or random coil environments, but that we cannot exclude the occurrence of PPII environment within the predicted random coils. Amino-acid quantification can take into account overlapping peaks only if they involve the same kind of residue, while two different residue overlapping peaks cannot be used using our quantification methodology (**Supplementary Table 10**). By analogy with glycan quantification, integrals are normalized using the number of carbon integrated (*tot nC*) and, for comparison with other residues, we take into account the number of spin system assigned (*n spin syst.*). Results of the HPLC-based quantification are shown for comparison in the last column.

		sum int.	nC	nC overlap	tot nC	int/nC_{total}	n spin syst.	(int/nC)*$n_{spin\ system}$	% norm.	HPLC
Ala	TOTAL	3.4E+08	30	8	38	8.9E+06	14	1.2E+08	13.03	5.30
	c random	1.6E+08	12	6	18	8.8E+06	5	4.4E+07	4.64	
	b beta	1.8E+08	18	2	20	9.0E+06	9	8.1E+07	8.52	
Gln	TOTAL	3.6E+08	18	4	22	1.6E+07	5	8.2E+07	8.59	8.10
	c random	9.8E+07	10	0	10	9.8E+06	2	2.0E+07	2.06	
	b beta	2.6E+08	8	4	12	2.2E+07	3	6.6E+07	6.95	
Glu	TOTAL	4.5E+08	20	0	20	2.3E+07	4	9.0E+07	9.40	6.70
	c random	3.5E+08	12	0	12	2.9E+07	2	5.8E+07	6.12	
	b beta	1.0E+08	8	0	8	1.3E+07	2	2.5E+07	2.66	
Asp	TOTAL	1.8E+08	13	0	13	1.4E+07	4	5.6E+07	5.82	9.00
	c random	1.4E+08	9	0	9	1.5E+07	3	4.6E+07	4.83	
	b beta	4.3E+07	4	0	4	1.1E+07	1	1.1E+07	1.14	
Gly	TOTAL	1.6E+08	10	0	10	1.6E+07	5	8.1E+07	8.46	8.00
	c random	1.5E+08	8	0	8	1.9E+07	4	7.5E+07	7.88	
	b beta	1.2E+07	2	0	2	6.1E+06	1	6.1E+06	0.64	
Asn	TOTAL	2.4E+08	12	0	12	2.0E+07	3	6.1E+07	6.36	1.00
	c random	2.4E+08	12	0	12	2.0E+07	3	6.1E+07	6.41	
	b beta	0.0E+00	0	0	0	0.0E+00	0	0.0E+00	0.00	
Val	TOTAL	2.3E+08	19	5	24	9.4E+06	6	5.6E+07	5.88	4.80
	c random	3.0E+07	6	0	6	5.0E+06	2	1.0E+07	1.06	
	b beta	2.0E+08	13	5	18	1.1E+07	4	4.3E+07	4.56	
Leu	TOTAL	3.2E+08	26	2	28	1.1E+07	7	7.9E+07	8.22	3.50
	c random	1.7E+08	18	0	18	9.3E+06	5	4.7E+07	4.91	
	b beta	1.5E+08	8	2	10	1.5E+07	2	2.9E+07	3.09	
Lys	TOTAL	2.1E+08	14	0	14	1.5E+07	4	5.9E+07	6.15	5.00
	c random	0.0E+00	0	0	0	0.0E+00	0	0.0E+00	0.00	

	b beta	2.1E+08	14	0	14	1.5E+07	4	5.9E+07	6.19	
Ser	TOTAL	2.4E+08	18	2	20	1.2E+07	5	5.9E+07	6.18	4.60
	c random	1.5E+08	12	0	12	1.3E+07	3	3.8E+07	4.03	
	b beta	8.4E+07	6	2	8	1.0E+07	2	2.1E+07	2.20	
Pro	TOTAL	3.9E+08	29	6	35	1.1E+07	5	5.6E+07	5.80	8.00
	c random	3.9E+08	29	6	35	1.1E+07	5	5.6E+07	5.84	
	b beta	0.0E+00	0	0	0	0.0E+00	0	0.0E+00	0.00	
Thr	TOTAL	2.2E+08	14	2	16	1.4E+07	3	4.1E+07	4.23	4.60
	c random	0.0E+00	0	0	0	0.0E+00	0	0.0E+00	0.00	
	b beta	2.2E+08	14	2	16	1.4E+07	3	4.1E+07	4.26	
Arg	TOTAL	7.6E+07	11	0	11	6.9E+06	7	4.8E+07	5.05	2.40
	c random	2.7E+07	3	0	3	9.1E+06	2	1.8E+07	1.91	
	b beta	4.9E+07	8	0	8	6.1E+06	5	3.1E+07	3.21	
Ile	TOTAL	1.0E+08	9	11	20	5.1E+06	4	2.0E+07	2.13	2.00
	c random	0.0E+00	0	0	0	0.0E+00	0	0.0E+00	0.00	
	b beta	1.0E+08	9	11	20	5.1E+06	4	2.0E+07	2.15	
Phe	TOTAL	4.5E+07	11	2	13	3.5E+06	2	7.0E+06	0.73	5.50
	c random	4.5E+07	11	2	13	3.5E+06	2	7.0E+06	0.73	
	b beta	0.0E+00	0	0	0	0.0E+00	0	0.0E+00	0.00	
Tyr	TOTAL	1.1E+08	17	23	40	2.9E+06	5	1.4E+07	1.49	1.60
	c random	1.1E+08	17	23	40	2.9E+06	5	1.4E+07	1.51	
	b beta	0.0E+00	0	0	0	0.0E+00	0	0.0E+00	0.00	
His	TOTAL	0.0E+00	0	0	0	0.0E+00	0	0.0E+00	0.00	5.60
	c random	0.0E+00	0	0	0	0.0E+00	0	0.0E+00	0.00	
	b beta	0.0E+00	0	0	0	0.0E+00	0	0.0E+00	0.00	
Met	TOTAL	1.8E+07	3	0	3	5.9E+06	4	2.4E+07	2.48	3.00
	c random	1.8E+07	3	0	3	5.9E+06	4	2.4E+07	2.50	
	b beta	0.0E+00	0	0	0	0.0E+00	0	0.0E+00	0.00	
Hyp	TOTAL	0.0E+00	0	0	0	0.0E+00	0	0.0E+00	0.00	2.80
	c random	0.0E+00	0	0	0	0.0E+00	0	0.0E+00	0.00	
	b beta	0.0E+00	0	0	0	0.0E+00	0	0.0E+00	0.00	
Cys	TOTAL	0.0E+00	0	0	0	0.0E+00	0	0.0E+00	0.00	0.20
	c random	0.0E+00	0	0	0	0.0E+00	0	0.0E+00	0.00	
	b beta	0.0E+00	0	0	0	0.0E+00	0	0.0E+00	0.00	

Supplementary Table 12. Amino acid composition of *wt* and *bald2 C. reinhardtii* strains. These quantities are obtained by HPLC from cell-wall extracts. Amino acids highlighted in orange are the amino acids that can be involved in O- or N-glycosylation. Therefore, standard deviation given in the last column represent a statistical analysis over 4 different samples.

Amino acid	wt-1 Relative mol (%)	wt-2 Relative mol (%)	bald2-1 Relative mol (%)	bald2-2 Relative mol (%)	Relative mole (%) avg	**stdDev**
**Asx (Asn+Asp)**	15.0	15.2	14.6	14.5	14.8	**0.3**
**Glx(Glu+Gln)**	18.9	18.6	19.4	19.2	19.0	**0.3**
**Hydroxyproline**	1.0	1.2	0.8	1.1	1.0	**0.1**
**Serine**	5.2	5.2	4.9	4.8	5.0	**0.2**
**Glycine**	8.5	7.8	7.7	8.1	8.0	**0.3**
**Histidine**	1.9	2.2	1.8	2.0	2.0	**0.1**
**Arginine**	3.6	3.7	3.0	3.5	3.5	**0.3**
**Threonine**	4.8	4.2	5.3	4.6	4.7	**0.4**
**Alanine**	10.5	11.0	10.8	11.2	10.9	**0.3**
**Proline**	5.0	4.7	4.5	5.0	4.8	**0.2**
**Tyrosine**	2.0	2.6	2.5	2.3	2.4	**0.2**
**Valine**	6.0	5.2	5.7	5.6	5.6	**0.3**
**Methionine**	1.7	1.4	2.0	1.4	1.6	**0.2**
**Isoleucine**	2.6	2.9	3.5	3.2	3.1	**0.3**
**Leucine**	5.1	5.8	5.4	5.8	5.5	**0.3**
**Phenylalanine**	2.9	3.2	2.7	2.2	2.8	**0.4**
**Lysine**	5.3	5.1	5.4	5.5	5.3	**0.1**

Supplementary Table 13. Protein-glycan contacts detected by MAS-DNP in the cell wall. MAS-DNP ^{13}C-^{13}C DARR spectra allows identification of contacts between hydroxyproline/threonine (red and green values, respectively, on the left table) and surrounding glycans. Despite low MAS-DNP resolution, specific glycan to amino acid contacts could be detected with Hyp or Thr (red and green values, respectively, on the right table) or both (bold underlined values).

Amino acid	δ_1 (ppm)	AA
Hyp	58.8	**Hypa**
	34.8	**Hypb**
	73.1	**Hypc**
	50.2	**Hypd**
Thr	19.6	**Thrc**
	19.4	**Thrc**
	17.5	**Thrc**

Sugar unit	C_1	C_2	C_3	C_4	C_5	C_6
Man1	104.5	73.9	74.9	**81.7**	77.2	61.8
Man2	**97.7**	74.6	76.2	65.7	77.2	61.4
Man3	100.8	71.5	73.6	67.8	77.1	61.8
Man4	**103.6**	72.2	75.4	67.9	77.2	61.9
Man5	**100.2**	76.8	71.4	68.4	77.7	65.7
Man6	**102.4**	67.8	77.2	65.7	76.0	62.1
Man7	**101.7**	67.9	77.1	65.6	76.0	62.1
Man8	**103**	71.1	77.1	67.8	75.3	63.1
Man9	**94.6**	69.9	78.1	68.3	74.1	64.9
Man10	96.7	70.4	76.8	65.8	76.0	62.2
Man11	97.6	68.6	80	66.2	76.9	61.9
Man12	96.8	68.6	**80.8**	66.3	76.9	61.5
Man13	**94.5**	70.2	78.6	66.7	77	61.8
Man14	**99.2**	73.6	78.5	65.9	70.5	66.2
Ara1	**110.3**	**82.4**	77.6	**81.8**	61.8	
Ara2	**110.1**	**82.2**	77.7	**81.7**	61.8	
Ara3	**109.8**	**81.9**	77.2	**81.4**	66.1	
Ara4	**109.8**	**81.8**	75	**81.3**	67.2	
Gal1	**103.5**	72.1	75.4	67.7	72.2	61.8
Gal2	**98.5**	72.4	73.7	71.7	75.1	62.9
Gal3	**105.8**	73.3	75	**81.1**	74.8	64.8
Rha1	**100.4**	77.1	70.2	71.7	70.3	17.8
Rha2	**100.4**	77.1	70.2	71.9	69.8	17.6
Rha3	**100.3**	70.2	**78.7**	73.6	70.9	17.4
Glc1	**100.4**	69.6	74.1	72.5	70.9	63.6
Glc2	**100**	76.8	71.3	63.8	71.8	61.9
Xyl1	97.7	76.1	77.2	67.7	60.2	
Xyl2	**100.3**	70.8	75.9	74.8	64.9	
Xyl3	**100.9**	77.4	75.1	**79.5**	67.8	

Supplementary Table 14. Glycoproteomics and identification of hexose modified proteins/peptides. These proteins/peptides were identified using ConA-LWAC enrichment. The accession number is based on taxonomy ID 3A3052_29 downloaded from Uniprot (May 13, 2023), and the table includes identifications for *Chlamydomonas reinhardtii* only. The "Modification" column indicates number of hexoses identified on each peptide; the specific site is indicated for specific entries based on the reliability of MS/MS data. Mass accuracy of precursor ions and deviation (ppm) from theoretical glycopeptide mass is indicated in the last column.

Protein	Peptide	Accession (Uniprot)	Modification	Activation	Charge	m/z	Δ ppm
**Guanylate cyclase domain-containing protein**	VLSLASNDLVGTLPHALSR	A0A2K3DIV2	T12(Hex)	ETciD	3	709.05511	0.37
**Guanylate cyclase domain-containing protein**	VLSLASNDLVGTLPHALSR	A0A2K3DIV2	T12(Hex)	ETciD	3	709.05505	0.28
**Guanylate cyclase domain-containing protein**	VLSLASNDLVGTLPH	A0A2K3DIV2	T12(Hex)	ETciD	2	849.45160	0.15
**Guanylate cyclase domain-containing protein**	VLSLASNDLVGTLPHAL	A0A2K3DIV2	T12(Hex)	ETciD	2	941.51227	0.21
**Guanylate cyclase domain-containing protein**	VLSLASNDLVGTLPH	A0A2K3DIV2	T12(Hex)	ETciD	2	849.45227	0.94
**Guanylate cyclase domain-containing protein**	VLSLASNDLVGTLPHALS	A0A2K3DIV2	T12(Hex)	ETciD	2	985.02826	0.18
**Guanylate cyclase domain-containing protein**	VLSLASNDLVGTLPHALSR	A0A2K3DIV2	(Hex)	HCD	3	709.05511	0.37
**Guanylate cyclase domain-containing protein**	VLSLASNDLVGTLPHALSR	A0A2K3DIV2	(Hex)	HCD	3	709.05505	0.28
**Guanylate cyclase domain-containing protein**	VLSLASNDLVGTLPH	A0A2K3DIV2	(Hex)	HCD	2	849.45160	0.15
**Guanylate cyclase domain-containing protein**	VLSLASNDLVGTLPHALS	A0A2K3DIV2	(Hex)	HCD	2	985.02856	0.49
**Guanylate cyclase domain-containing protein**	VLSLASNDLVGTLPHAL	A0A2K3DIV2	(Hex)	HCD	2	941.51227	0.21
**Guanylate cyclase domain-containing protein**	VLSLASNDLVGTLPH	A0A2K3DIV2	(Hex)	HCD	2	849.45227	0.94
**Guanylate cyclase domain-containing protein**	VLSLASNDLVGTLPHALS	A0A2K3DIV2	(Hex)	HCD	2	985.02826	0.18
**Guanylate cyclase domain-containing protein**	VLSLASNDLVGTLPHAL	A0A2K3DIV2	(Hex)	HCD	2	941.51245	0.40
**Leucine-rich repeat-containing N-terminal plant-type domain-containing protein**	ISGTLPPSWQSWQSVR	A0A2K3D0L8	(Hex)	HCD	2	995.99768	0.57
**Leucine-rich repeat-containing N-terminal plant-type domain-containing protein**	DNSFSGTLPDSWQLLR	A0A2K3DTP7	(Hex)	HCD	2	999.47589	-0.33
**Leucine-rich repeat-containing N-terminal plant-type domain-containing protein**	NPNIAGSLPPEWAAGL	A0A2K3DTP7	S7(Hex)	HCD	2	884.94159	0.34
**Leucine-rich repeat-containing N-terminal plant-type domain-containing protein**	DNSFSGTLPDSWQLLR	A0A2K3DTP7	(Hex)	HCD	2	999.47614	-0.08
**Leucine-rich repeat-containing N-terminal plant-type domain-containing protein**	LVGTVPEVLGSLPYLSR	A0A2K3DN96	(Hex)	HCD	2	981.54352	0.15
**Oxygen-evolving enhancer protein 2, chloroplastic**	VTNKSGFVPYAGDGFALLLPAK	P11471	S5(Hex-Hex)	ETciD	3	863.78308	0.46
**Oxygen-evolving enhancer protein 2, chloroplastic**	HQLIGATVGSDNKLYIIK	P11471	S10(Hex-Hex)	ETciD	3	765.40918	0.16
**Protein kinase domain-containing protein**	HLVLPAATK	A0A835SF10	T8(Hex-Hex)	HCD	2	637.34888	1.50

**Uncharacterized protein**	SGTLPALPTSLQVLR	A0A2K3DV32	(Hex)	HCD	3	572.32440	0.02
**Uncharacterized protein**	SSNSFSGTLPALPTSLQVLR	A0A2K3DV32	(Hex)	HCD	2	1119.08630	-0.32
**Uncharacterized protein**	SNSFSGTLPALPTSLQVLR	A0A2K3DV32	(Hex)	HCD	2	1075.57080	0.14
**Uncharacterized protein**	NSLSGTLPR	A0A2K3DV32;A0A835TDM1	(Hex)	HCD	2	553.78815	0.16

Supplementary Table 15. ^{13}C DARR cross-peak intensities and calculated hydration level. Resonance-specific control and water-edited spectra (**Supplementary Fig. 12**) were used for this hydration level calculation.

Spin system	Resolved peaks	S/S0	error bar	average/unit
Lys	Lys-c-1-b/c	0.23	0.12	
	Lys-c-1-e/d	0.14	0.05	
	Lys-c-2-a/b	0.28	0.12	
	Lys-c-2-b/c	0.07	0.03	
	Lys-c-2-e/d	0.05	0.02	
	Lys-c-3-b/c	0.08	0.03	
	Lys-c-4-b/c	0.10	0.05	0.14
Asp	Asp-b-1-c/b	0.02	0.01	
	Asp-b-2-c/b	0.01	0.01	
	Asp-c-3-c/b	0.07	0.02	
	Asp-a-4-a/b	0.02	0.01	
	Asp-a-4-c/b	0.04	0.01	0.03
Asn	Asn-b-1-c/b	0.20	0.09	
	Asn-b-1-o/a	0.22	0.07	
	Asn-b-2-a/b	0.13	0.05	
	Asn-b-2-c/b	0.34	0.14	
	Asn-b-2-o/a	0.22	0.07	
	Asn-c-3-c/b	0.06	0.02	0.20
Glu	Glu-c-1-a/b	0.03	0.01	
	Glu-c-1-d/c	0.04	0.01	
	Glu-c-1-o/a	0.06	0.03	
	Glu-a-2-a/b	0.06	0.03	
	Glu-a-2-d/c	0.05	0.01	
	Glu-c-3-d/c	0.03	0.01	
	Glu-c-4-a/b	0.02	0.01	
	Glu-c-4-c/b	0.06	0.02	
	Glu-c-4-d/c	0.07	0.02	0.05
Gln	Gln-b-1-c/b	0.01	0.02	
	Gln-b-1-d/c	0.09	0.17	
	Gln-b-2-d/c	0.05	0.02	
	Gln-c-3-d/c	0.04	0.07	
	Gln-b-4-a/b	0.07	0.02	
	Gln-b-4-c/b	0.02	0.01	
	Gln-b-4-d/c	0.03	0.01	
	Gln-b-5-c/b	0.07	0.02	
	Gln-b-5-d/c	0.04	0.01	
	Gln-b-5-o/a	0.03	0.01	0.05

Pro	Pro-c-1-b/c	0.07	0.03	
	Pro-c-1-d/c	0.06	0.02	
	Pro-c-1-o/a	0.11	0.04	
	Pro-c-2-a/b	0.01	0.03	
	Pro-c-2-b/c	0.02	0.01	
	Pro-c-2-d/c	0.03	0.06	
	Pro-b-3-b/c	0.08	0.03	
	Pro-b-3-d/c	0.09	0.04	
	Pro-b-3-o/a	0.22	0.00	
	Pro-c-4-a/b	0.05	0.02	
	Pro-c-4-b/c	0.00	0.00	
	Pro-c-4-d/c	0.02	0.01	
	Pro-c-5-a/b	0.02	0.01	
	Pro-c-5-b/c	0.03	0.01	
	Pro-c-5-d/c	0.13	0.05	
	Pro-c-5-o/a	0.18	0.09	0.07
Arg	Arg-b-1-b/c	0.17	0.05	
	Arg-b-2-d/c	0.11	0.04	
	Arg-b-3-b/c	0.09	0.03	
	Arg-c-5-b/c	0.07	0.03	
	Arg-c-6-b/c	0.16	0.06	0.12
Gly	Gly-b-1-o/a	0.04	0.01	
	Gly-c-2-o/a	0.02	0.01	
	Gly-c-3-o/a	0.03	0.01	
	Gly-c-4-o/a	0.02	0.01	
	Gly-a-5-o/a	0.03	0.01	0.03
Ala	Ala-b-1-a/b	0.06	0.02	
	Ala-b-2-a/b	0.03	0.01	
	Ala-b-3-a/b	0.09	0.03	
	Ala-b-4-a/b	0.02	0.01	
	Ala-b-5-a/b	0.13	0.06	
	Ala-b-6-a/b	0.05	0.02	
	Ala-c-7-a/b	0.01	0.00	
	Ala-c-9-a/b	0.02	0.01	
	Ala-c-10-a/b	0.00	0.00	
	Ala-c-10-o/a	0.01	0.00	
	Ala-c-11-a/b	0.01	0.00	
	Ala-c-11-o/a	0.10	0.04	
	Ala-c-12-a/b	0.07	0.02	
	Ala-c-13-a/b	0.14	0.05	
	Ala-c-14-a/b	0.17	0.05	0.06
Tyr	Tyr-b-1-c/b	0.05	0.02	

	Tyr-b-1-d/c	0.06	0.03	
	Tyr-b-1-d/e	0.04	0.01	
	Tyr-b-1-f/e	0.14	0.04	
	Tyr-b-2-f/e	0.19	0.08	
	Tyr-b-3-c/b	0.05	0.02	
	Tyr-b-3-d/c	0.06	0.02	
	Tyr-b-3-d/e	0.18	0.07	
	Tyr-b-3-f/e	0.16	0.05	
	Tyr-b-4-c/b	0.05	0.02	
	Tyr-b-4-d/c	0.06	0.03	
	Tyr-b-4-d/e	0.04	0.02	
	Tyr-b-4-f/e	0.19	0.07	
	Tyr-b-5-c/b	0.05	0.01	
	Tyr-b-5-d/c	0.06	0.02	
	Tyr-b-5-d/e	0.18	0.08	
	Tyr-b-5-f/e	0.02	0.01	0.09
Leu	Leu-a-1-a/b	0.10	0.04	
	Leu-a-1-b/c	0.07	0.02	
	Leu-a-1-c/d	0.15	0.05	
	Leu-a-2-c/d	0.07	0.04	
	Leu-a-3-a/b	0.09	0.03	
	Leu-a-3-c/d	0.08	0.03	
	Leu-c-5-a/b	0.05	0.02	
	Leu-c-6-b/c	0.02	0.01	
	Leu-c-6-o/a	0.02	0.01	
	Leu-c-7-b/c	0.15	0.04	
	Leu-c-7-c/d	0.07	0.03	
	Leu-c-7-o/a	0.02	0.01	0.08
Met	Met-c-1-c/d	0.12	0.04	0.12
Val	Val-c-1-o/a	0.16	0.06	
	Val-a-2-a/b	0.01	0.01	
	Val-a-2-b/c	0.10	0.03	
	Val-a-3-a/b	0.11	0.04	
	Val-c-4-a/b	0.01	0.00	
	Val-c-4-b/c	0.07	0.02	
	Val-c-5-a/b	0.02	0.01	
	Val-c-5-b/c	0.07	0.03	
	Val-c-6-a/b	0.02	0.01	
	Val-c-6-o/a	0.02	0.01	0.06
Ile	Ile-c-1-a/b	0.05	0.02	
	Ile-c-1-c1/d	0.03	0.01	
	Ile-c-2-b/c2	0.06	0.02	
	Ile-c-3-c1/d	0.04	0.01	

	Ile-u-4-b/c	0.06	0.03	0.05
Phe	Phe-u-1-c/d	0.10	0.05	
	Phe-u-1-e/f	0.05	0.02	
	Phe-u-2-c/d	0.10	0.03	
	Phe-u-2-d/e	0.08	0.04	
	Phe-u-2-e/f	0.02	0.01	0.07
Hyp	Hyp-u-a/b	0.86	0.16	
	Hyp-u-c/b	0.84	0.17	
	Hyp-u-c/d	0.85	0.16	0.85
Ser	Ser-b-1-b/a	0.40	0.08	
	Ser-b-1-o/a	0.41	0.09	
	Ser-b-2-b/a	0.23	0.05	
	Ser-b-2-o/a	0.31	0.07	
	Ser-b-3-b/a	0.31	0.07	
	Ser-c-4-b/a	0.33	0.08	
	Ser-c-4-o/a	0.27	0.06	
	Ser-c-5-b/a	0.42	0.10	
	Ser-c-5-o/a	0.50	0.11	0.35
Thr	Thr-b-1-b/c	0.32	0.07	
	Thr-b-2-b/a	0.47	0.10	
	Thr-b-2-b/c	0.50	0.10	
	Thr-b-2-o/a	0.49	0.09	
	Thr-b-3-b/a	0.58	0.11	
	Thr-b-3-b/c	0.46	0.08	
	Thr-b-3-o/a	0.57	0.13	0.49
GlcNAc	Ac-1	0.21	0.05	
	Ac-2	0.13	0.03	
	Ac-3	0.16	0.04	
	Ac-4	0.22	0.05	0.18
Ara	Ara-1-2/3	0.37	0.09	
	Ara-2-4/5	0.46	0.09	
	Ara-3-1/2	0.66	0.14	
	Ara-4-4/5	0.19	0.04	0.42
Gal	Gal-2-1/2	0.90	0.21	
	Gal-2-3/2	0.81	0.18	
	Gal-3-1/2	0.74	0.15	
	Gal-3-4/5	0.81	0.18	
	Gal-3-5/6	0.84	0.19	0.82

Glc	Glc-1-1/2	0.46	0.11	
	Glc-1-3/4	0.36	0.08	
	Glc-1-4/5	0.51	0.11	
	Glce-2-1/2	0.41	0.08	
	Glce-2-3/4	0.37	0.08	0.42
Man	Man-1-1/2	0.25	0.06	
	Man-1-4/5	0.72	0.16	
	Man-1-5/6	0.54	0.11	
	Man-2-1/2	0.53	0.13	
	Man-3-1/2	0.73	0.17	
	Man-3-5/6	0.91	0.20	
	Man-4-1/2	0.78	0.18	
	Man-4-3/2	0.87	0.19	
	Man-4-5/4	0.79	0.16	
	Man-5-1/2	0.34	0.08	
	Man-5-2/3	0.32	0.07	
	Man-6-3/2	0.79	0.17	
	Man-6-3/4	0.69	0.15	
	Man-6-5/6	0.18	0.04	
	Man-7-1/2	0.67	0.14	
	Man-7-3/2	0.74	0.15	
	Man-8-3/4	0.79	0.19	
	Man-8-5/6	0.49	0.11	
	Man-9-1/2	0.41	0.09	
	Man-9-3/2	0.24	0.05	
	Man-9-3/4	0.27	0.06	
	Man-9-5/4	0.26	0.06	
	Man-10-1/2	0.35	0.08	
	Man-10-3/2	0.75	0.18	
	Man-11-3/2	0.39	0.08	
	Man-11-3/4	0.70	0.16	
	Man-11-5/6	0.66	0.14	
	Man-12-3/2	0.41	0.10	
	Man-12-5/4	0.66	0.16	
	Man-13-3/2	0.31	0.07	
	Man-14-3/2	0.36	0.08	0.55
Rha	Rha-1-5/6	0.26	0.05	
	Rha-2-1/2	0.59	0.14	
	Rha-2-5/6	0.37	0.09	
	Rha-3-1/2	0.54	0.13	
	Rha-3-5/6	0.38	0.09	0.43
Xyl	Xyl-1-1/2	0.52	0.11	
	Xyl-1-3/2	0.60	0.14	

	Xyl-2-1/2	0.56	0.12	
	Xyl-2-3/4	0.37	0.08	
	Xyl-3-2/3	0.59	0.13	
	Xyl-3-4/3	0.45	0.10	
	Xyl-3-4/5	0.68	0.16	0.54

Supplementary Table 16. Resonance-specific ^{13}C intensity DNP build-up. Intensities are measured on 1D 13C MAS-DNP spectra at 600 MHz/395 GHz, using AMUPol as polarizing agent and 8 kHz MAS frequency. Intensities are indicated as a function of DNP microwave (MW) time and plotted on **Supplementary Fig. 13**. Tentative assignment is given but the resolution is not as good as on 2D spectra.

	Assignment	^{13}C δ(ppm) \ MW time	0.1	0.2	0.4	0.8	1.6	3.2	6.4	12.8	25.6
Amino acids	Ile C_γ	15.6	3.4	6.8	14.6	26.2	45.3	67.8	88.0	98.4	100.0
	Leu C_δ	21.7	3.2	7.3	14.9	28.1	48.5	72.9	91.8	98.9	100.0
	Ile C_γ/Pro C_γ/Ala C_β/Arg C_γ	25.2	2.8	6.4	13.6	26.3	46.1	71.2	90.9	98.5	100.0
	Gln C_β	29.9	2.8	7.0	13.8	26.6	46.4	71.5	91.7	99.5	100.0
	Glu C_γ/Hyp C_β/Glu C_γ	34.7	3.0	6.8	14.2	26.5	46.9	71.9	92.2	99.3	100.0
	Leu C_β	39.8	2.7	6.8	13.4	26.1	46.2	71.4	91.8	99.3	100.0
	Ala C_α	49.6	2.8	6.9	14.3	26.4	45.8	70.6	90.5	99.3	100.0
	Asn C_α	52.9	3.0	6.6	13.8	26.1	46.3	71.0	91.2	99.0	100.0
	Ala C_o	173.5	3.1	7.0	14.2	27.0	47.7	73.5	92.9	99.6	100.0
	Hyp C_α	58.7	3.3	7.3	14.9	**28.1**	**48.7**	73.5	92.3	99.4	100.0
	glycan C_6/ Val Cα	62.5	3.4	7.6	15.4	**29.1**	**50.0**	74.7	93.1	99.2	100.0
glycans	Man $C_{4/6}$	65.8	3.6	7.9	15.8	**29.6**	**50.4**	75.1	93.1	99.1	100.0
	Man C_2/Rha C_5/Glc C_2	69.8	3.6	8.0	15.8	**29.8**	**50.5**	75.3	93.2	99.2	100.0
	Gal C_2	73.0	3.6	7.9	15.8	**30.0**	**51.1**	75.7	93.6	99.3	100.0
	Ara C_2	82.3	4.4	8.2	16.6	**30.7**	**53.1**	78.3	97.0	99.9	100.0
	Gal C_1	98.5	3.7	8.4	16.1	**30.4**	**51.3**	75.9	94.5	98.9	100.0

Supplementary Table 17. Assignment of 1D INEPT and CP ^{13}C ssNMR peaks.

Amino acids

^{13}C δ (ppm)	Assignment	INEPT	CP
54.1	Asp4Cα	x	
179.1	Glu2Co		x
30.1	Glu2Cγ		x
28.3	Glu4Cβ	x	
179.0	Gln1Cδ		x
30.5	Gln5Cβ		x
58.8	Hyp1Cα	x	
50.2	Hyp1Cδ	x	
59.2	Pro3Cα	x	
31.5	Arg1Cβ	x	
30.4	Arg2Cβ		x
55.8	Arg5Cα	x	
173.9	Thr2Co		x
43.6	Gly4Cα		x
17.3	Ala13Cβ	x	
51.1	Tyr4Cα		x
130.2	Tyr5Cγ	x	
23.0	Leu7Cδ	x	
53.8	Met3Cα	x	
172.4	Val6Co		x
11.1	Ile1Cδ	x	
173.7	Ile3Co		x
16.3	Ile4Cγ2		x
128.1	Phe2Cε	x	

Glycans

^{13}C δ (ppm)	Assignment	INEPT	CP
81.7	Man1C4	x	
77.2	Man1C5	x	
61.4	Man2C6	x	
103	Man8C1		x
67.8	Man8C4		x
69.9	Man9C2		x
96.7	Man10C1	x	
97.6	Man11C1		x
80.8	Man12C3		x
94.5	Man13C1		x
67.7	Gal1C4	x	
105.8	Gal3C1		x
81.1	Gal3C4		x
69.8	Rha2C5		x
73.6	Rha3C4		x
69.6	Glc1C2		x
74.1	Glc1C3		x
67.7	Xyl1C4	x	
100.3	Xyl2C1	x	
74.8	Xyl2C4	x	

Supplementary Table 18. ^{13}C resonance CP intensity build-up in *C. reinhardtii*'s cell wall extract. Tentative assignments are given but one-dimensional experiments involve peak overlapping compared to 2D spectra.

	Assignment	^{13}C δ (ppm) \ CP (ms)	**0.001**	**0.01**	**0.05**	**0.1**	**0.2**	**0.3**	**0.5**	**0.75**	**1**	**1.5**	**2**	**3**	**4**	**5**	**7.5**	**10**
amino acids	Ile C_γ	15.4	0.00	0.05	0.21	0.44	0.86	0.89	1.00	0.92	0.93	0.89	0.86	0.78	0.70	0.63	0.51	0.41
	Ile C_γ	16.3	0.00	0.02	0.23	0.45	0.88	0.87	1.00	0.89	0.95	0.88	0.85	0.76	0.69	0.61	0.52	0.42
	Met C_δ/Val C_γ/Ala C_β	19.2	0.01	0.03	0.20	0.32	0.85	0.93	1.00	0.95	0.98	0.93	0.89	0.81	0.73	0.66	0.51	0.42
	Leu C_δ	21.7	0.01	0.04	0.29	0.39	0.83	0.93	1.00	0.99	0.98	0.95	0.90	0.81	0.74	0.64	0.51	0.30
	Arg C_γ/Pro C_γ	24.9	0.01	0.08	0.42	0.50	0.96	0.96	1.00	0.99	0.97	0.92	0.87	0.76	0.67	0.61	0.48	0.28
	Val C_β/Met C_γ	29.7	0.01	0.11	0.27	0.47	0.84	0.95	1.00	0.98	0.96	0.91	0.85	0.75	0.67	0.60	0.49	0.39
	Hyp C_β/Glu C_γ	35.0	0.01	0.19	0.34	0.62	0.88	0.96	1.00	0.95	0.90	0.84	0.78	0.70	0.63	0.57	0.46	0.36
	Arg C_δ/Ile C_β	37.9	0.02	0.16	0.39	0.61	0.77	0.93	1.00	0.96	0.91	0.85	0.78	0.67	0.60	0.54	0.43	0.36
	Ala C_α	50.0	0.01	0.16	0.29	0.59	0.78	0.92	1.00	0.94	0.88	0.85	0.80	0.72	0.65	0.58	0.47	0.40
	Val C_α/Pro C_α	61.4	0.01	0.05	0.30	0.32	0.65	0.75	0.81	0.89	0.94	0.99	1.00	0.94	0.85	0.79	0.64	0.53
	Phe C_ε	128.6	0.01	0.05	0.45	0.60	0.82	0.95	1.00	0.96	0.88	0.84	0.77	0.63	0.58	0.54	0.43	0.31
	C_o	172.2	0.00	0.01	0.10	0.33	0.50	0.66	0.78	0.92	0.97	0.97	1.00	0.97	0.90	0.79	0.64	0.49
Glycans	glycan C_6	63.2	0.01	0.06	0.37	0.52	0.82	0.94	1.00	0.97	0.98	0.99	0.97	0.95	0.89	0.83	0.73	0.66
	Glc C_2/Rha C_5/Man C_2	69.7	0.01	0.09	0.47	0.64	0.85	0.91	0.97	1.00	1.00	0.99	0.97	0.90	0.82	0.76	0.63	0.54
	Gal C_2	73.2	0.01	0.09	0.47	0.64	0.84	0.90	0.94	0.97	0.98	1.00	0.98	0.93	0.85	0.79	0.66	0.58
	Man C_3	76.4	0.01	0.07	0.35	0.48	0.62	0.75	0.83	0.89	0.93	0.99	1.00	0.97	0.88	0.82	0.67	0.57
	Ara C_2	82.1	0.02	0.10	0.57	0.78	0.88	0.98	1.00	0.97	0.96	0.93	0.91	0.83	0.77	0.71	0.60	0.51
	Ara C_2	83.9	0.01	0.11	0.57	0.73	0.91	0.99	1.00	0.95	0.93	0.90	0.87	0.82	0.73	0.67	0.54	0.45
	Man C_1	94.8	0.00	0.14	0.53	0.70	0.80	0.94	0.99	0.99	1.00	0.97	0.94	0.85	0.78	0.72	0.56	0.46
	Man/Xyl C_1	97.1	-0.01	0.05	0.33	0.46	0.53	0.69	0.79	0.88	0.96	1.00	0.98	0.86	0.79	0.71	0.53	0.43
	Gal C_1	98.2	0.00	0.12	0.58	0.80	0.83	1.00	1.00	0.97	0.96	0.95	0.91	0.83	0.75	0.69	0.57	0.49
	Man/Rha/Xyl/Rha/Glc C_1	100.2	0.02	0.09	0.47	0.63	0.74	0.87	0.92	0.96	0.98	1.00	0.98	0.90	0.84	0.79	0.65	0.56
	Man/Gal C_1	103.6	-0.01	0.04	0.29	0.37	0.62	0.79	0.88	0.99	0.98	1.00	0.94	0.82	0.70	0.62	0.46	0.32
	Ara C_1	109.4	-0.02	0.09	0.46	0.69	0.89	0.93	1.00	1.00	0.98	0.96	0.97	0.92	0.81	0.74	0.64	0.57
	Ara C_1	108.5	-0.01	0.08	0.45	0.66	0.82	0.96	1.00	0.94	0.94	0.93	0.88	0.79	0.73	0.65	0.52	0.43

Supplementary Table 19. ^{13}C resonance CP intensity build-up in *C. reinhardtii*'s starch. Resonance-specific ^{13}C intensity build-up as a function of cross-polarization (CP) time in starch purified from *C. reinhardtii* [15]. Assignments are given as described before[15].

		^{13}C δ (ppm) \ CP (ms)	0.01	0.025	0.05	0.075	0.1	0.25	0.5	0.75	1	2	3	5
C. reinhardtii A-type starch	C1 am	102.6	0.12	0.29	0.52	0.83	1.00	1.00	1.00	0.76	0.72	0.60	0.56	0.48
	C1a	101.2	0.13	0.36	0.60	0.92	0.97	0.94	1.00	0.97	0.96	0.92	0.86	0.80
	C1b	100.6	0.12	0.33	0.57	0.86	1.00	0.96	0.96	0.93	0.96	0.93	0.87	0.81
	C1c	99.5	0.12	0.33	0.55	0.87	1.00	0.93	0.98	0.89	0.88	0.87	0.82	0.76
	C4 am	80.8	0.14	0.34	0.53	0.80	0.98	1.00	0.90	0.84	0.71	0.60	0.54	0.47
	C3	75.4	0.12	0.33	0.56	0.85	1.00	0.97	0.99	1.00	0.97	0.87	0.77	0.71
	C3	74.1	0.11	0.30	0.54	0.82	1.00	0.97	0.97	0.94	0.91	0.80	0.72	0.68
	C5	72.1	0.10	0.28	0.48	0.76	0.92	1.00	0.99	0.92	0.93	0.86	0.81	0.74
	C6	61.7	0.12	0.31	0.53	0.80	0.93	0.97	1.00	0.93	0.93	0.86	0.77	0.69

Supplementary Table 20. List of experiments performed in this work and acquisition time. Key parameters used for acquisition are also presented.

	Interest	Technique	Temperature (K)	Spinning frequency (kHz)	Initial polarization	Experiment	Mixing time	Recycling delay (s)	Number of scans	Number of increments	Total time (h)
**Cell-wall extract**	Composition & Structure	ssNMR 800 MHz	283	13.5	DP	INADEQUATE + THALOS$^+$		2	64	350	12.6
	Dynamics & dynamic filters	ssNMR 600 MHz	293	15	CP	^{13}C $T_{1\rho}$ (us)		3	32	12	0.3
					CP	^{1}H $T_{1\rho}$ (us)		3	32	12	0.3
					DP	^{13}C T_1 (us)		10	32	17	1.5
					CP	^{1}H T_1 (us)		10	32	19	1.7
		ssNMR 800 MHz	283	13.5	DP	1D DP 30s		30	128		0.0
					DP	1D DP 2 s		2	128		0.0
					CP	1D CP		2	128		0.0
					INEPT	1D INEPT		4	128		0.0
					CP	INADEQUATE		1.5	64	200	5.4
					CP	DARR		1.75	32	600	9.4
		ssNMR 600 MHz	293	15	CP	DARR	25 ms	2.5	64	256	11.4
					CP	PDSD	250 ms	2.5	32	256	5.7
					CP	DARR	300 ms	3	32	256	6.9
					CP	PDSD	1 s	3	32	256	6.9
					long CP (10 ms)	PDSD	1 s	3	32	256	6.9
					long CP (10 ms)	DARR	300 ms	3	32	256	6.9
					CP + ^{13}C $T_{1\rho}$ filter	DARR	25 ms	3	64	256	13.7
					CP + ^{13}C $T_{1\rho}$ filter	PDSD	250 ms	3	69	256	14.8
		ssNMR 600 MHz at cryogenic temperature	98	8	CP	DARR	50 ms	2.86	16	512	6.6
	Deglycosylation	ssNMR 600 MHz	278	15	CP	DARR	50 ms	2.5	64	256	11.4

	Hydration	ssNMR 800 MHz	283	13.5	CP	DARR	50 ms	1.75	32	600	9.4
					CP & water edition	DARR	50 ms	1.75	32	600	9.4
	Protein-glycan contacts	MAS-DNP 600 MHz □w on	100	8	CP	DARR	50 ms	2.86	16	512	6.6
					CP	PDSD	1.5 s	2.86	16	400	5.1
					DP	INADEQUATE		2.99	16	533	7.1
					CP	NCa	0	2.8	16	533	6.7
					CP	NCaCx	100 ms	2.8	16	533	6.7
		ssNMR 800 MHz	283	13.5	CP	NCa	0	1.5	48	180	3.6
					CP	NCaCx	100 ms	1.5	48	180	3.6
		ssNMR 600 MHz	278	15	CP	DARR	300 ms	3	32	256	6.9
					CP	PDSD	1 s	3	32	256	6.9
**Whole cell**	Composition	ssNMR 800 MHz	283	13.5	DP	INADEQUATE		2	64	350	12.6
	Dynamic filters				CP	INADEQUATE		1.5	64	200	5.4
	Structure				CP	DARR	50 ms	1.75	32	600	9.4
	Hydration				CP & water edition	DARR	50 ms	1.75	32	600	9.4
	Structure	MAS-DNP 600 MHz □w on	100	8	CP	DARR	50 ms	2.86	16	512	6.6

Supplementary Table 21. List of replicates used for ssNMR in this study. All the replicates showed identical overall composition and architecture based on 1D experiments (DP with 2 and 30 s recycling delay, CP and INEPT pulse sequences) that were ran on all the samples. Replicates consist in different cell culture leading to different cell-wall extracts. It must be noted that part of each culture was kept before cell-wall extraction for whole cell monitoring. Interestingly, cell-wall extraction done on cw15 cell-wall deficient strain did not give any material after dialysis.

Strain used for cell-wall extracts	Number of biological replicates
Wild-type (CC-124)	6
Wild-type - deglycosylated	2
Flagella deficient bald2	6
Flagella deficient bald2 - deglycosylated	2
Cell-wall deficient cw15	2 (no cw extract)
Mating types CC-124 (“mt+”)	2
Mating types CC-125 (“mt-”)	2
Starchless sta 6-1	2

References

1. Vila, J. A. & Arnautova, Y. A. in *Computational Methods to Study the Structure and Dynamics of Biomolecules and Biomolecular Processes: From Bioinformatics to Molecular Quantum Mechanics* (ed Adam Liwo) 659-698 (Springer International Publishing, 2019).
2. Fujiwara, K., Toda, H. & Ikeguchi, M. Dependence of α-helical and β-sheet amino acid propensities on the overall protein fold type. *BMC Struct. Biol.* **12**, 18 (2012).
3. Ferris, P. J. *et al.* Glycosylated polyproline II rods with kinks as a structural motif in plant hydroxyproline-rich glycoproteins. *Biochemistry* **40**, 2978-2987 (2001).
4. Homer, R. B. & Roberts, K. Glycoprotein conformation in plant cell walls : circular dichroism reveals a polyproline II structure. *Planta* **146**, 217-222 (1979).
5. Voigt, J. & Frank, R. 14-3-3 proteins are constituents of the insoluble glycoprotein framework of the *Chlamydomonas* cell wall. *Plant Cell* **15**, 1399-1413 (2003).
6. Shen, Y., Delaglio, F., Cornilescu, G. & Bax, A. TALOS+: a hybrid method for predicting protein backbone torsion angles from NMR chemical shifts. *J. Biomol. NMR* **44**, 213-223 (2009).
7. Schäler, K. *Low-fiield NMR studies of structure and dynamics of semicrystalline polymers* Doctoral thesis thesis, Martin-Luther-Universität Halle-Wittenberg, (2013).
8. Mathieu-Rivet, E., Mati-Baouche, N., Walet-Balieu, M.-L., Lerouge, P. & Bardor, M. N- and O-glycosylation pathways in the microalgae polyphyletic group. *Front. Plant Sci.* **11** (2020).
9. Lesage, A., Sakellariou, D., Steuernagel, S. & Emsley, L. Carbon−proton chemical shift correlation in solid-state NMR by through-bond multiple-quantum spectroscopy. *J. Am. Chem. Soc.* **120**, 13194-13201 (1998).
10. Gao, Y., Lipton, A. S., Wittmer, Y., Murray, D. T. & Mortimer, J. C. A grass-specific cellulose–xylan interaction dominates in sorghum secondary cell walls. *Nat. Commun.* **11**, 6081 (2020).
11. Simmons, T. J. *et al.* Folding of xylan onto cellulose fibrils in plant cell walls revealed by solid-state NMR. *Nat. Commun.* **7**, 13902 (2016).
12. Zhao, W. *et al.* Solid-state NMR of unlabeled plant cell walls: high-resolution structural analysis without isotopic enrichment. *Biotechnol. Biofuels* **14**, 14 (2021).
13. Kirui, A. *et al.* Carbohydrate-aromatic interface and molecular architecture of lignocellulose. *Nat. Commun.* **13**, 538 (2022).
14. Kang, X. *et al.* Molecular architecture of fungal cell walls revealed by solid-state NMR. *Nat. Commun.* **9**, 2747 (2018).
15. Poulhazan, A., Arnold, A. A., Warschawski, D. E. & Marcotte, I. Unambiguous *ex situ* and in Cell 2D ^{13}C solid-state NMR characterization of starch and its constituents. *Int. J. Mol. Sci.* **19**, 3817 (2018).
16. Poulhazan, A. *et al.* Identification and quantification of glycans in whole cells: architecture of microalgal polysaccharides described by solid-state nuclear magnetic resonance. *J. Am. Chem. Soc.* **143**, 19374-19388 (2021).
17. Gorin, P. A. J. & Mazurek, M. Further Studies on the Assignment of Signals in ^{13}C Magnetic Resonance Spectra of Aldoses and Derived Methyl Glycosides. *Can. J. Chem.* **53**, 1212-1223 (1975).
18. Bradbury, J. H. & Jenkins, G. A. Determination of the structures of trisaccharides by ^{13}C-n.m.r. spectroscopy. *Carbohydr. Res.* **126**, 125-156 (1984).
19. Lundborg, M. & Widmalm, G. Structural analysis of glycans by NMR chemical shift prediction. *Anal. Chem.* **83**, 1514-1517 (2011).
20. Hwang, J.-H. *et al.* Photoautotrophic hydrogen production by eukaryotic microalgae under aerobic conditions. *Nat. Commun.* **5**, 3234 (2014).
21. White, P. B., Wang, T., Park, Y. B., Cosgrove, D. J. & Hong, M. Water–polysaccharide interactions in the primary cell wall of *Arabidopsis thaliana* from polarization transfer solid-state NMR. *J. Am. Chem. Soc.* **136**, 10399-10409 (2014).
22. Phyo, P. *et al.* Gradients in wall mechanics and polysaccharides along growing inflorescence stems. *Plant Physiol.* **175**, 1593 (2017).
23. Rondeau-Mouro, C., Buléon, A. & Lahaye, M. Caractérisation par RMN des biopolymères d'origine végétale, de la molécule à l'organisation supramoléculaire. *Comptes rendus Chimie* **11**, 370-379 (2008).
24. King, C., Stein, R. S., Shamshina, J. L. & Rogers, R. D. Measuring the purity of chitin with a clean, quantitative solid-state NMR method. *ACS Sustain. Chem. Eng.* **5**, 8011-8016 (2017).
25. Habibi, Y., Heyraud, A., Mahrouz, M. & Vignon, M. R. Structural features of pectic polysaccharides from the skin of *Opuntia ficus*-indica prickly pear fruits. *Carbohydr. Res.* **339**, 1119-1127 (2004).

26. Laguri, C. *et al.* Solid State NMR Studies of Intact Lipopolysaccharide Endotoxin. *ACS Chem. Biol.* **13**, 2106-2113 (2018).
27. Pfeffer, P. E., Hicks, K. B., Frey, M. H., Opella, S. J. & Earl, W. L. Complete solid state ^{13}C NMR chemical shift assignments for α-D-glucose, α-D-glucose-H_2O and β-D-glucose. *J. Carbohydr. Chem.* **3**, 197-217 (1984).
